# Synthesis, X-ray Structure, Hirshfeld, DFT Conformational, Cytotoxic, and Anti-Toxoplasma Studies of New Indole-Hydrazone Derivatives

**DOI:** 10.3390/ijms241713251

**Published:** 2023-08-26

**Authors:** Eman M. Hassan, Saied M. Soliman, Esraa A. Moneer, Mohamed Hagar, Assem Barakat, Matti Haukka, Hanaa Rasheed

**Affiliations:** 1 Department of Chemistry, Faculty of Science, Alexandria University, P.O. Box 426, Ibrahimia, Alexandria 21321, Egypt; emymohamed201435@gmail.com (E.M.H.); saeed.soliman@alexu.edu.eg (S.M.S.); 2 Department of Medical Laboratory Technology, Faculty of Applied Health Sciences Technology, Pharos University in Alexandria, Alexandria 21500, Egypt; esraa.moneer@pua.edu.eg; 3 Department of Chemistry, College of Science, King Saud University, P.O. Box 2455, Riyadh 11451, Saudi Arabia; ambarakat@ksu.edu.sa; 4 Department of Chemistry, University of Jyväskylä, P.O. Box 35, FI-40014 Jyväskylä, Finland; matti.o.haukka@jyu.fi

**Keywords:** indole-hydrazone, conformational analysis, X-ray structure, cytotoxic, anti-toxoplasma, *T. gondii*

## Abstract

The hydrazones **3a**–**c**, were synthesized from the reaction of indole-3-carbaldehyde and nicotinic acid hydrazide, isonicotinic acid hydrazide, and benzoic acid hydrazide, respectively. Their structures were confirmed using FTIR, ^1^HNMR, and ^13^CNMR spectroscopic techniques. Exclusively, hydrazones **3b** and **3c** were confirmed using single crystal X-ray crystallography to exist in the ***Eanti*** form. With the aid of DFT calculations, the most stable configuration of the hydrazones **3a**–**c** in gas phase and in nonpolar solvents (CCl_4_ and cyclohexane) is the ***ESyn*** form. Interestingly, the DFT calculations indicated the extrastability of the ***EAnti*** in polar aprotic (DMSO) and polar protic (ethanol) solvents. Hirshfeld topology analysis revealed the importance of the N…H, O…H, H…C, and π…π intermolecular interactions in the molecular packing of the studied systems. Distribution of the atomic charges for the hydrazones **3a**–**c** was presented. The hydrazones **3a**–**c** showed a polar character where **3b** has the highest polarity of 5.7234 Debye compared to the **3a** (4.0533 Debye) and **3c** (5.3099 Debye). Regarding the anti-toxoplasma activity, all the detected results verified that **3c** had a powerful activity against chronic toxoplasma infection. Compound **3c** showed a considerable significant reduction percent of cyst burden in brain homogenates of toxoplasma infected mice representing 49%.

## 1. Introduction

Hydrazones are a class of compounds present in many of the bioactive heterocyclic compounds that are very important because of their biological and clinical applications. For example, hydrazone-based coupling methods are used in medical biotechnology to couple drugs to target antibodies, e.g., antibodies against a certain type of tumor cell. The hydrazones are stable at neutral pH but are rapidly destroyed in the acidic environment around the tumor, and hence designing a pH-sensitive drug delivery system is an advantage. These characteristics have allowed hydrazones to be linked to many drugs for many applications [1,2,3,4,5,6,7,8,9]. Additionally, hydrazones are good chelating agents due to their ability to form stable complexes with many transition metals. These metal complexes have potential applications as catalysts, luminescent probes, and molecular sensors [1,7]. The tridentate coordination mode of the hydrazone ligands make them suitable for bis-chelating mono-nucleating agents for metal ions.

Moreover, many reports showed an activity of hydrazones as antiprotozoal agents. Siddiqui et al. [10] synthesized aryl hydrazone derivatives and tested them for anti-amoebic activity against the HM1: IMSS strain of Entamoebica histolytica and they were found to have an IC_50_ of 0.13. M. Gerpe et al. [11] reported that 5-nitrofuran hydrazones show anti-Trypanosomal action. Caputto et al. [12] synthesized hydrazine derivatives and reported anti-*Trypanosoma cruzi* (*T. cruzi*) activity. Cinnamic N-acyl hydrazones with anti-Trypanosomal action were synthesized by Carvalho et al. [13]. Dos Santos Filho et al. [14] prepared hydrazone derivatives to combat T. Trypanosomicidal activity of hydrazones [15]. The reported inhibitory activity of fused aromatic hydrazones against cruzipain-a major cysteine protease of *T. cruzi* was reported [16].

A variety of hydrazone derivatives have been prepared to alleviate gastrointestinal discomfort and toxicity. Mohamed Eissa et al. [17] synthesized anthranilic acid compounds that were shown to have strong anti-inflammatory action. Hydrazones comprising 5-methyl-2-benzoxazolinones designed by Salgin-Gökşen et al. [18] have been shown to have a good analgesic and anti-inflammatory effect. Khan et al. [19] described the anti-inflammatory action of quinoxalinone hydrazone derivatives.

Specifically, Toxoplasmosis is a global neglected tropical parasitic disease, caused by the obligate intracellular apicomplexan protozoan, *Toxoplasma gondii*. The most important risk factors associated with *T. gondii* infection are handling cats, ingestion of undercooked meat, and also blood transfusion [20]. *T. gondii* infection can encyst chronically in the brain leading to a broad spectrum of neurological complications. The majority of toxoplasmosis cases are due to reactivation of existing chronic infections. All therapeutic drugs currently available are effective only against tachyzoites and have limited efficacy against tissue cysts so it is necessary to investigate a new appropriate drug that can effectively reduce brain cyst burden [21].

Density functional theory (DFT) calculations are a low-cost computational method to predict molecular structure and predict the relative stability of different isomers of organic compounds [22,23,24,25,26,27]. In this regard, the aim of the present work is to synthesize a new set of hydrazones derived from indole. Their molecular structure aspects were investigated using different experimental and theoretical tools. In addition, their biological activity as an anti-parasitic agent was assessed.

## 2. Results and Discussion

### 2.1. Synthesis of the Acid Hydrazides and Characterization

**Scheme 1:**
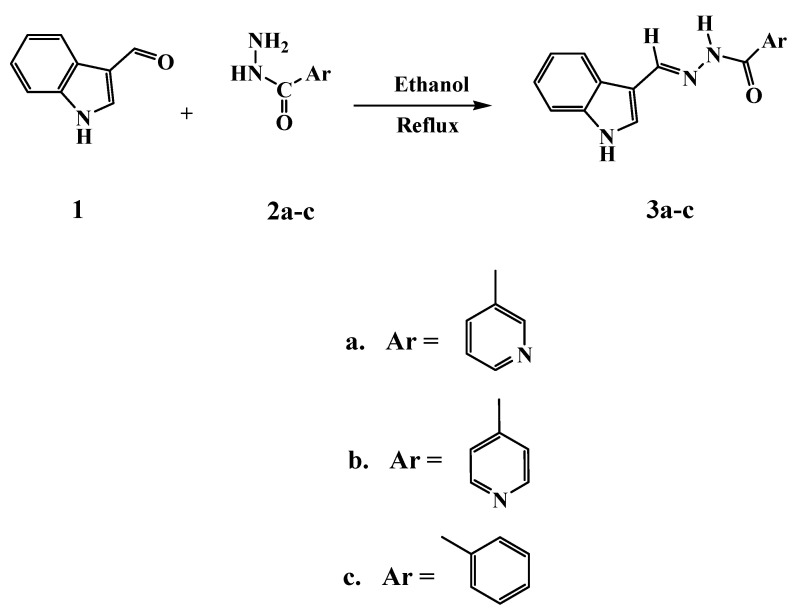
Preparation of the investigated hydrazones **3a**–**c**.

The investigated acid hydrazides were synthesized according to Scheme 1. The condensation reaction of the indole-3-carbaldehyde (**1**) and the molar equivalent appropriate acid hydrazide (**2a**–**c**) (nicotinic acid hydrazide, isonicotinic acid hydrazide, and benzoic acid hydrazide) afforded the corresponding hydrazones (**3a**–**c**) in good yields (61−81%). The structure of the investigated compounds was confirmed by the spectral data. The ^1^H NMR showed the presence of NH groups as four singles at the chemical shift of 11.5 ppm. Moreover, the carbonyl groups were approved in terms of the stretching vibration at 1640−1648 cm^−1^.

**Scheme 2:**
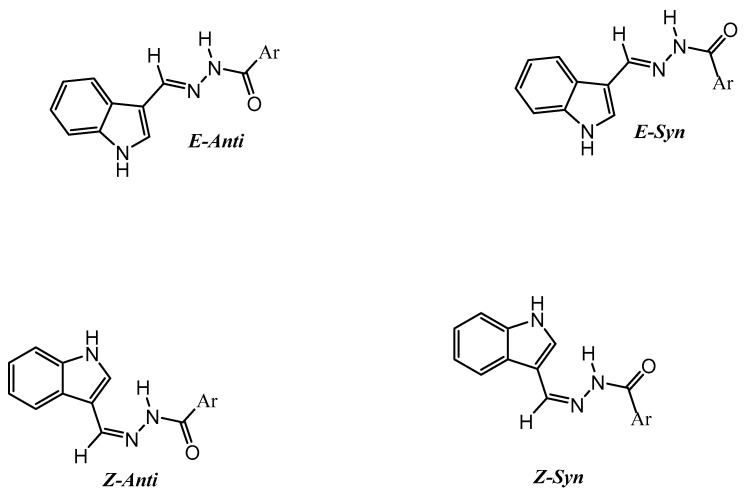
The isomeric forms of the investigated hydrazones **3b** and **3c**.

According to Scheme 2, acylhydrazones could exist in equilibrium mixture of the various isomeric forms, *Zanti*, *Eanti*, *Zsyn*, and *Esyn*. 

**Scheme 3:**
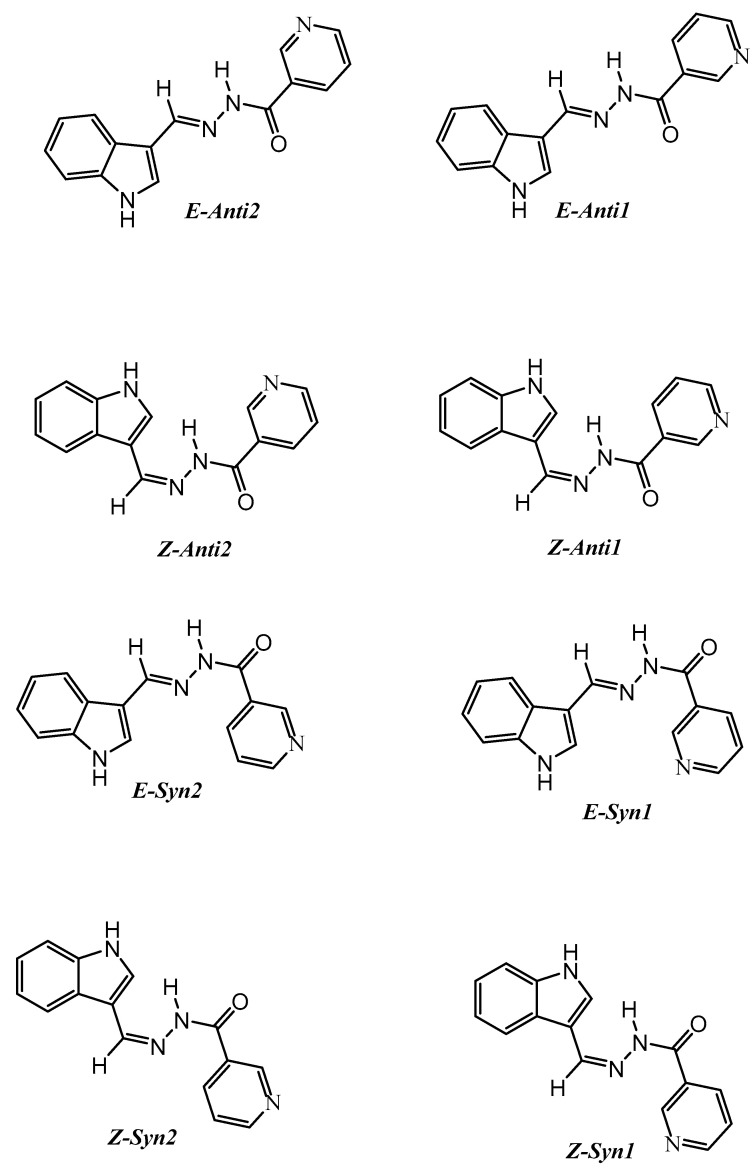
The isomeric forms of the investigated hydrazone **3a**.

On the other hand, to deeply study the effect of orientation of the pyridyl group on the stability of the studied hydrazones, more isomers have been studied in terms of the orientation of the pyridyl group of compound **3a**, as clearly shown in Scheme 3.

### 2.2. X-ray Structure Description

**Figure 1:**
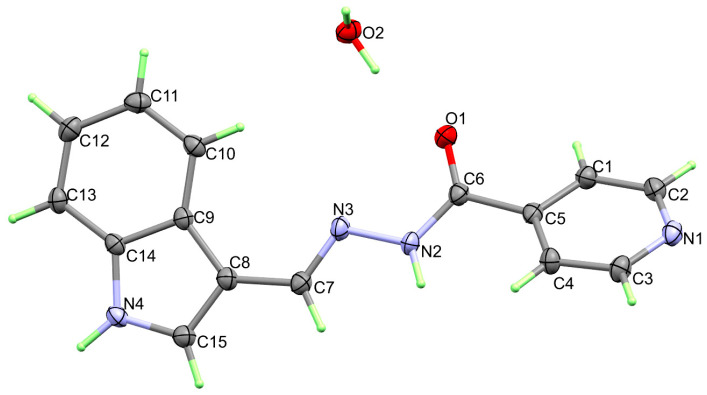
Thermal ellipsoids at 30% probability level showing atom numbering of **3b**.

The X-ray structure of **3b** indicated with no doubt the presence of this compound in the ***EAnti*** configuration in the solid state and no evidence for other conformers was detected (Figure 1). A list of bond distances and angles is given in Table 1. The **3b** is found to be crystallized in the monoclinic crystal system and space group of *P2**_1_**/c*. The unit cell parameters are *a* = 7.04998(8), *b* = 25.0269(3), *c* = 7.88162(9), and *β* = 104.165(11)°. In this case, the asymmetric formula comprised one molecule of the organic target compound and one crystal water molecule. Moreover, there are four molecules of this asymmetric formula per unit cell where its volume is 2672.31(3) Å^3^. The two aromatic ring systems are found twisted from one another, where the twist angle between the two ring systems is 45.68°.

**Figure 2:**
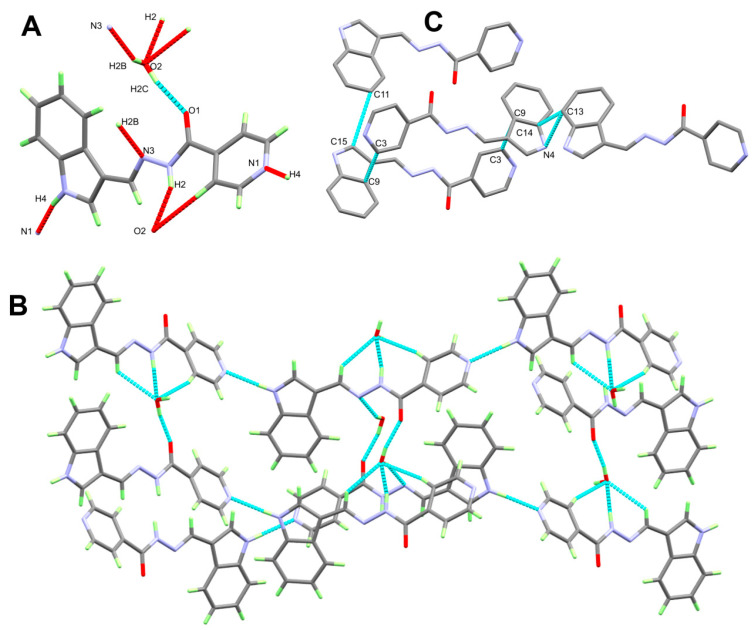
The different contacts and the supramolecular structure of **3b**.

The supramolecular structure of **3b** is controlled by strong N2-H2…O2, O2-H2C…O1 and N4-H4…N1 hydrogen bond contacts in addition to weak O2-H2B…N3 and C4-H4A…O2 interactions (Figure 2A). The hydrogen bond parameters of these non-covalent interactions are listed in Table 2 while the corresponding packing scheme is shown in Figure 2B. The donor to acceptor distances are 2.8903(12), 2.7705(11), 2.9120(11), 3.0927(12), and 3.4231(13) Å, respectively while the hydrogen-acceptor distances are 2.057(15), 1.886(15), 2.014(15), 2.283(17), and 2.52Å, respectively. In addition, the packing is further controlled by some π−π stacking interactions shown in Figure 2C. The C13…N4 (3.229(1) Å), C3…C9 (3.374(1) Å), C13…C14 (3.390(1) Å), and C11…C15 (3.257(1) Å) contacts are the shortest π−π contacts. 

**Figure 3:**
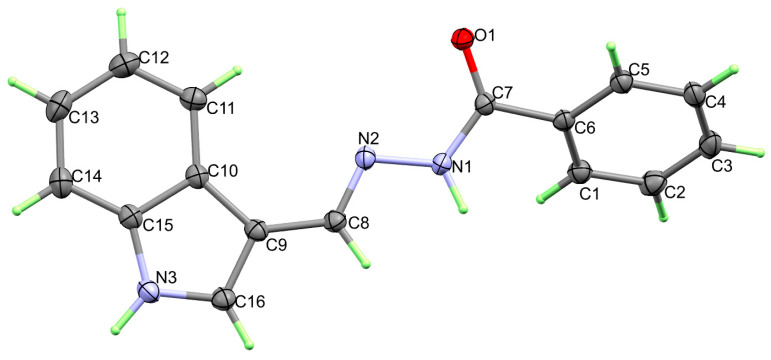
Thermal ellipsoids at 30% probability level showing atom numbering of **3c**.

The structure of **3c** and atom numbering are presented in Figure 3 while a list of bond distances and angles is given in Table 3. It crystallized in the more symmetric orthorhombic crystal system and the *Pbca* space group with z = 8 and one molecule as asymmetric formula. The unit cell parameters are *a* = 17.42273(12) Å, *b* = 7.69675(5) Å, *c* = 19.92797(14) Å, and 2672.31(3) Å^3^. There are two ring systems which are indole and phenyl moieties. It is clear that both ring systems are perfectly planar but are not coplanar to one another. The twist angle between the two ring systems is 52.19° which is relatively smaller than that in **3c**. It is clear from the X-ray structure that the compound **3c** is found in the ***EAnti*** configuration in the solid state and no evidence for other conformers was detected.

**Figure 4:**
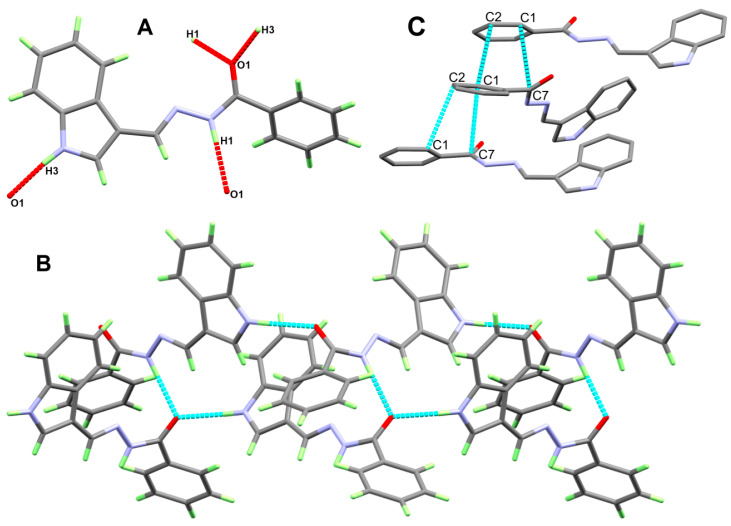
The different contacts and the supramolecular structure of **3c**.

The supramolecular structure of **3c** is controlled by strong N-H…O hydrogen bond contacts, as presented in Figure 4A. The hydrogen bond parameters are listed in Table 4 while the packing scheme via N1-H1…O3 and N3-H3…O3 is shown in Figure 4B. The donor (N1 and N3) to acceptor (O3) distances are 2.8827(11) and 2.8304(11) Å, respectively while the O3…H1 and O3…H3 distances are 1.981(15) and 1.936(16) Å, respectively. In addition, the packing is further controlled by some π−π stacking interactions shown in Figure 4C. The C1…C7 (3.353(1) Å) and C1…C2 (3.323(1) Å) contacts are the shortest π−π contacts.

### 2.3. Hirshfeld Analysis

**Figure 5:**
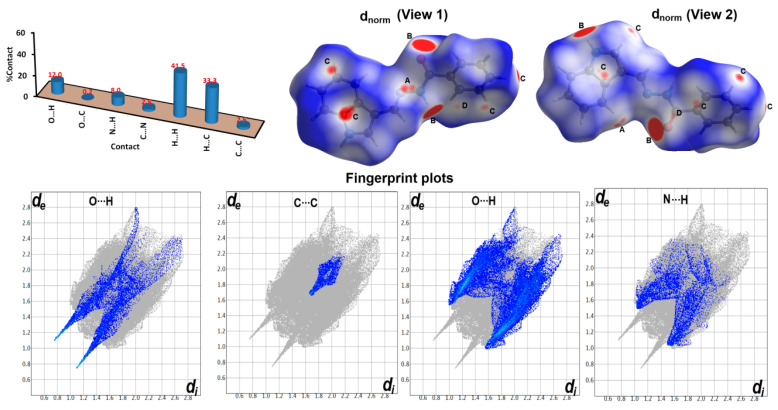
Hirshfeld analysis of **3c**; A: N…H, B: O…H, C: H…C, and D: C…C.

The molecular packing of **3c** is further investigated by using Hirshfeld calculations in order to shed light on all possible intermolecular contacts in its crystal structure. All possible contacts between molecular units along with their percentages are presented in Figure 5. The most common contacts are H…H (41.5%), H…C (33.3%), and O…H (12.0%) interactions. Other minor contact such as N…H (8.0%) and C…C (2.5%) interactions are also significant in the molecular packing of **3c**. The red spots in the d_norm_ are related to short intermolecular interactions, which are considered the most significant and also shorter than the van der Waals radius sum of the two atoms. The corresponding fingerprint plots shown in Figure 5 have the characteristic sharp spikes which are considered as another evidence for the short contacts. These contacts and their corresponding distances are listed in Table 5.

**Figure 6:**
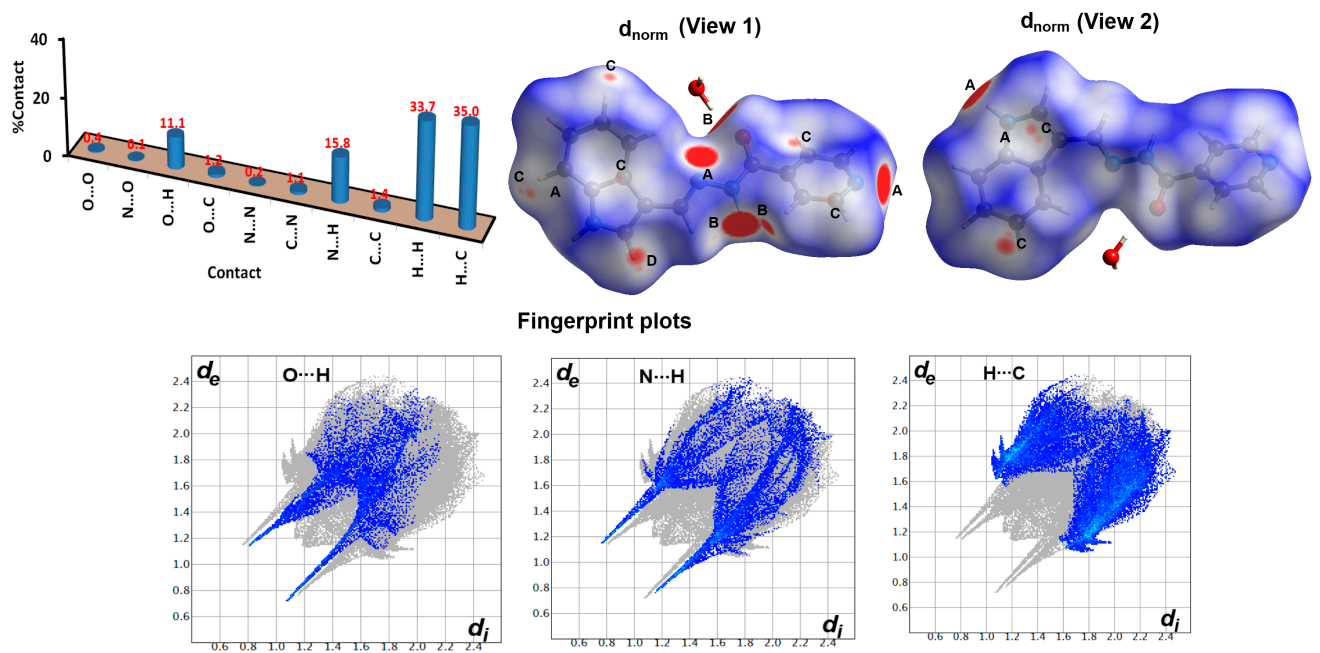
Hirshfeld analysis of **3b**; A: N…H, B: O…H, and C: H…C, and D: C…C.

Hirshfeld analysis of **3b** is presented in Figure 6. It is clear that the most dominant contacts are the H…H (33.7%), H…C (35.0%), N…H (15.8%), and O…H (11.1%) interactions. Other contacts shown in Figure 6 are contributing little and have less importance in the molecular packing of **3b**. In this crystal structure, the H…C, N…H and O…H interactions are the most important as all these contacts appeared as red spots in the d_norm_ map and also as sharp spikes in the corresponding fingerprint plots (Figure 6). The interaction distances of all possible short contacts in **3b** are collected in Table 6. In this case, the H…C interactions are weaker and have longer interaction distances (2.689–2.761 Å) compared to **3c** (2.557–2.785 Å). In contrast, the O…H and N…H interactions in **3b** occur at shorter distances compared to **3c**. The O1…H2C (1.802 Å) and N1…H4 (1.921 Å) in **3b** are shorter than those found in **3c** (O3…H1: 1.850 Å and N2…H11: 2.541 Å, respectively). In the case of **3b**, no short C…C contacts were detected, which indicates the low importance of the π−π stacking interactions. 

### 2.4. Conformational Analysis

#### 2.4.1. Energetic and Relative Stability

The structures of the different possible isomers of the studied compounds were calculated using a DFT/B3LYP method employing 6-31G(d,p) basis sets. The results of the energetic and thermodynamic parameters were used to investigate the relative stability of these isomers (Table 7). The detailed thermodynamic parameters are presented in Tables S1–S15 (Supplementary data). 

**Figure 7:**
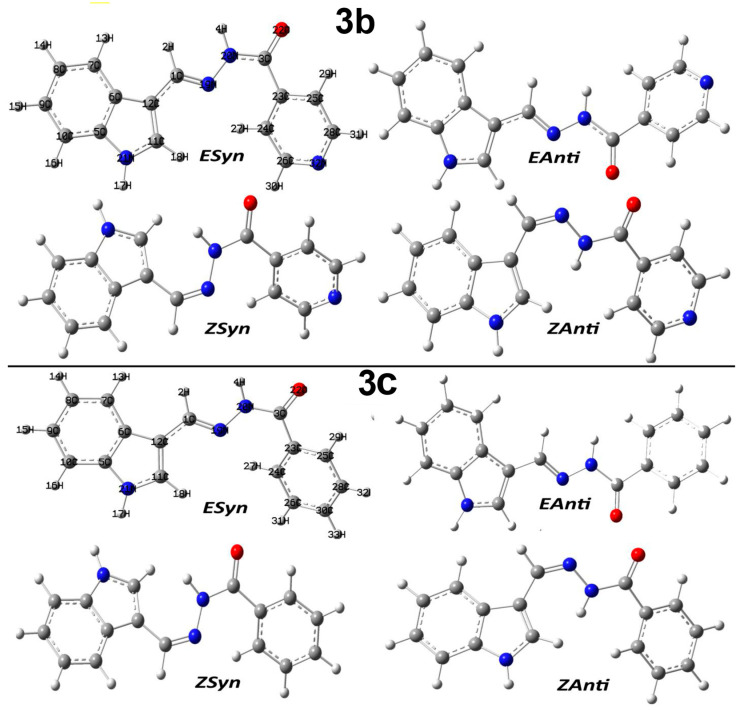
The optimized structures of the four possible conformers of **3b** and **3c**.

The optimized structures of the four possible isomers of compounds **3b** and **3c** in gas phase are shown in Figure 7. It is clear that the ***ESyn*** conformer is the most stable compared to the others. The second most stable form is ***EAnti***, which is energetically higher by 1.4964 and 1.2179 kcal/mol for **3b** and **3c**, respectively. Hence, the order of their stabilities is ***ESyn*** > ***EAnti*** > ***ZSyn*** > ***ZAnti***. Since the Enthalpy and Gibbs free energy of the ***ESyn*** form is the most negative, this isomer is the most stable form thermodynamically. The percentages of the most stable isomers ***ESyn*** and ***EAnti*** are calculated to be 90.8 and 7.2% for **3b** while the corresponding values for **3c** are 87.9 and 10.1%, respectively.

In order to inspect the relative stability of these isomers in different media, we calculated the four possible isomers in the presence of different solvents. In general, all isomers are stabilized by solvent effects as noticed from the lowering in their energies in solution compared to gas phase. It is clear that the polar solvents (ethanol and DMSO) stabilized these isomers to a higher extent compared to the nonpolar solvents (CCl_4_ and cyclohexane). As a result, there was no change in the order of the isomers’ stability in the case of nonpolar solvents where the ***ESyn*** is the most stable and the ***EAnti*** is the second most stable one. The energy differences between these isomers in the case of CCl_4_ as solvent are 0.7085 and 0.3803 Kcal/mol for **3b** and **3c**, respectively. The corresponding values in cyclohexane are 0.8066 and 0.4839 Kcal/mol, respectively. Hence, the order of the isomers’ stability is ***ESyn*** > ***EAnti*** > ***ZSyn*** > ***ZAnti***. The percentages of the most stable isomers ***ESyn*** and ***EAnti*** are calculated to be 72.79 and 26.14% for **3b** in CCl_4_. In cyclohexane, the corresponding values are 75.39 and 23.45%, respectively. Similar results were obtained for **3c** (Table 7). It is noticed that the %***ESyn*** isomer are decreased while %***EAnti*** are increased in presence of these solvents compared to those in gas phase.

Interestingly, the situation is completely revered in the case of polar solvents (DMSO and ethanol). In these solvents, the order of the stabilities is predicted to be ***EAnti*** > ***ESyn*** > ***ZAnti*** > ***ZSyn***. Hence, the ***EAnti*** isomer is predicted to be the most stable while ***ESyn*** is the second most stable form. In DMSO as polar aprotic solvent, the relative energies between the two isomers are calculated to be 0.8522 and 1.1399 Kcal/mol for **3b** and **3c**, respectively. For **3b**, the percentages of ***EAnti*** and ***ESyn*** are calculated to be 80.04 and 19.6%, respectively while the corresponding values for **3c** are 86.41 and 13.18%, respectively. The results are almost the same in ethanol as polar protic solvent (Table 7).

**Figure 8:**
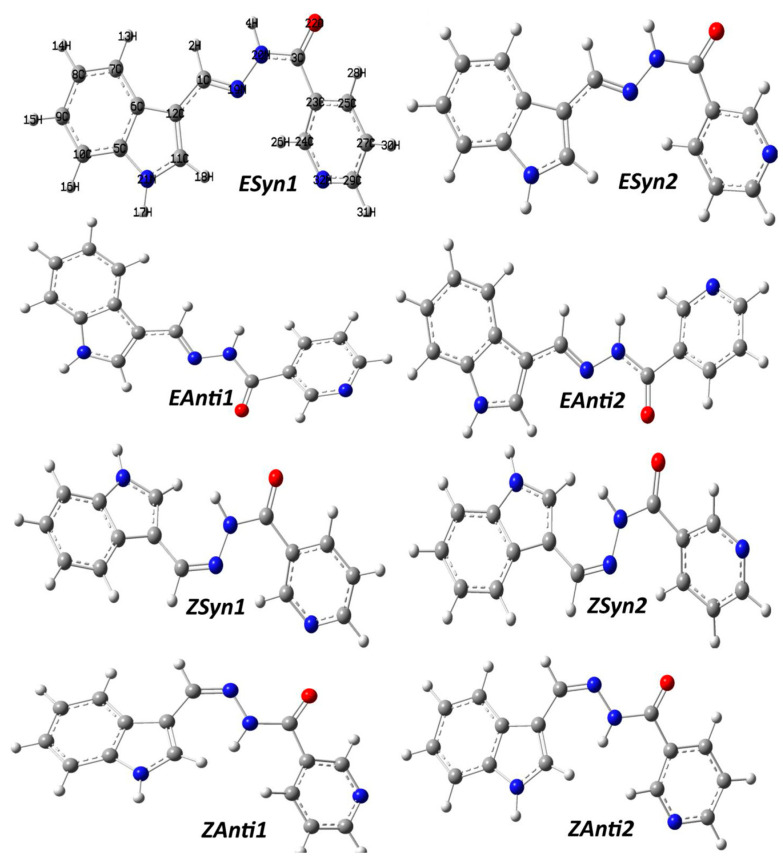
The optimized structures of the eight possible conformers of **3a**.

In case of **3a**, eight possible conformers were suggested. Their optimized structures are shown in Figure 8. Similar to **3b** and **3c**, ***ESyn*1** is the most stable while ***ESyn*2** is energetically marginally higher by 0.5733 kcal/mol. Hence, the ***ESyn*** isomers are considered the most stable in gas phase and the order of the isomers’ stabilities is ***ESyn*1** > ***ESyn*2** > ***EAnti*2** > ***ZSyn*1** > ***ZSyn*2** > ***EAnti*1** > ***ZAnti*1** > ***ZAnti*2.** These results are found in accord with the calculated thermodynamic parameters presented in Tables S11–S15 (Supplementary data). The net percentage of the most stable isomers ***ESyn*** is calculated to be 93.9%. Additionally, the stability order is predicted to be ***ESyn*1** > ***ESyn*2** > ***EAnti*2** > ***EAnti*1** > ***ZSyn*1** > ***ZSyn*2** > ***ZAnti*2** > ***ZAnti*1** in the presence of nonpolar solvents. It is clear that the ***ESyn*** is omers are the most stable forms and the energy difference between the two ***ESyn*** isomers is marginally different (Table 8). The net percentages of the ***ESyn*** isomers are 81.42 and 83.79% in CCl_4_ and cyclohexane, respectively. It is worth noting that the %***EAnti*** isomers are significantly increased compared to those in gas phase. In contrast, the polar solvents further stabilized the ***EAnti*** isomers compared to the ***ESyn*** ones. The ***EAnti*2** and ***EAnti*1** are the most stable forms where the former is energetically lower than the latter. The next two most stable isomers are ***ESyn*1** and ***ESyn*2** forms. In the case of DMSO as solvent, the percentages of these isomers are calculated to be 37.90, 29.33, 17.42, and 14.92%, respectively. The corresponding values for these isomers in ethanol are 35.87, 27.09, 19.69, and 16.81%, respectively.

#### 2.4.2. Natural Charge Analysis

**Figure 9:**
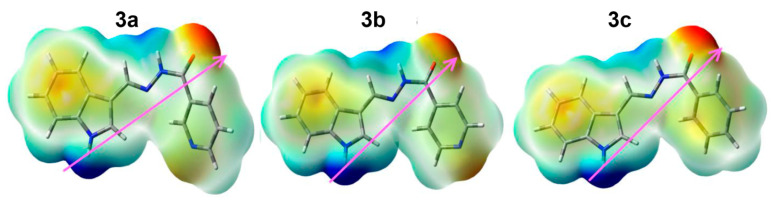
MEP maps for the studied systems; pink arrow for the direction of the dipole moment vector.

The charges at the different atomic sites of the most stable isomer (***ESyn***) were calculated based on natural population analysis and the results are presented in Table 9. For **3b**, the O22 (−0.603), N21 (−0.550), N20 (−0.446), and N32 (−0.440) atoms have the most negative charge, in agreement with the highly electronegative nature of the N and O-atoms. In contrast, the C3 (0.678), H17 (0.442), and H4 (0.403) atoms have the most positive charge, as these sites are directly attached to atomic sites with high electronegativity. Similarly for compound **3c**, the natural charge analysis indicated the high electronegative character of the carbonyl oxygen and amine nitrogen atoms. The highest negative charges are related to O22, N21, N20, and N32 which have natural charges of −0.611, −0.550, −0.446, and −0.446, respectively. As a result, the C3, H17, and H4 atoms have the highest positive charges of 0.677, 0.442, and 0.403, respectively. The corresponding charges of the electronegative atoms in the case of **3a** are −0.611 (O22), −0.550 (N21), −0.446 (N20), and −0.446 (N32) while the charges of the electropositive atoms C3, H17, and H4 are 0.677, 0.442, and 0.403, respectively. On the other hand, presentation of the different charged regions is shown in Figure 9. The molecular electrostatic potential (MEP) maps revealed with no doubt the high negative electron density related to the carbonyl oxygen atom, as indicated by the dark intense red color close to it. The dark blue areas related to the NH protons indicate the high positive charge density related to these sites. As a result of the different charged regions in the studied systems, the compounds **3a**, **3b**, and **3c** showed high polarity, as indicated from the high dipole moment values of 4.0533, 5.7234, and 5.3099 Debye, respectively.

### 2.5. Biology

#### 2.5.1. Cytotoxic Effects of the Studied Compounds

**Figure 10:**
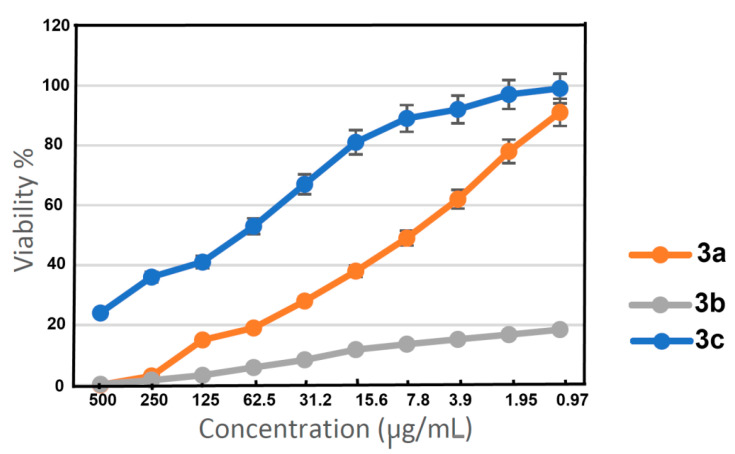
The percentage of normal fibroblast cells viability of **3a**–**c** at different levels of concentrations.

The in vitro cytotoxic effects of **3a**, **3b**, and **3c** were tested using a human lung fibroblast cell line (WI-38). The results are presented graphically in Figure 10. It was found that the fibroblast cell viability percentages were 41.0, 15.0, and 3.2% at 125 μg/mL for compounds **3c**, **3a**, and **3b,** respectively. Their IC_50_ values were 164, 48.25, and 11.03, respectively.

#### 2.5.2. Cyst Counts in Brain Homogenates

The mean cyst count in group II (infected non-treated) was 22.9 ± 1.7. The percentage of reduction in the **3c** treated group was the highest, representing 49%, and the mean cyst count was 11.6 ± 1.3, while reduction percentages in the **3a** and **3b** treated groups were 37% and 32%, respectively (Table 10).

#### 2.5.3. Morphological Changes

**Figure 11:**
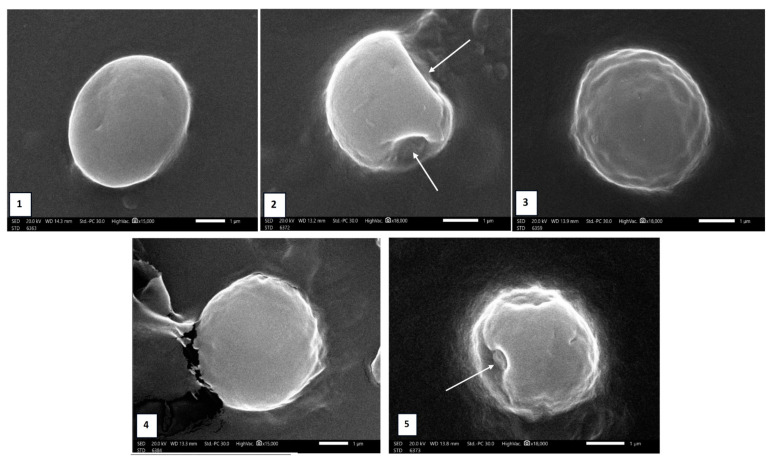
(**1**) SEM of *T. gondii* cysts of the infected control group (II) showed spherical shape with totally regular smooth surface. (**2**) Cysts from spiramycin treated group (III) showed total roughness and surface irregularities with retained spherical outlines and deep dimples (arrows). (**3**) Cysts from **3a** treated group (IV) showed an irregular outer surface. (**4**) Cyst from **3b** treated group (V) showed slight swelling and surface irregularities. (**5**) Cysts from **3c** treated group (VI) showed irregular spherical shape and cyst surface irregularities with a focal blebs formation (arrows).

The ultrastructural morphological features of *T. gondii* cysts in the mice brain homogenate for each studied group were evaluated using SEM. *T. gondii* cysts from the control group II seemed as regular spherical-shaped cysts with a regular cyst surface while cysts recovered from treated mice revealed variable degrees of morphological changes (Figure 11).

#### 2.5.4. Histopathological Examination

##### Cerebral Cortex of Brain

**Figure 12:**
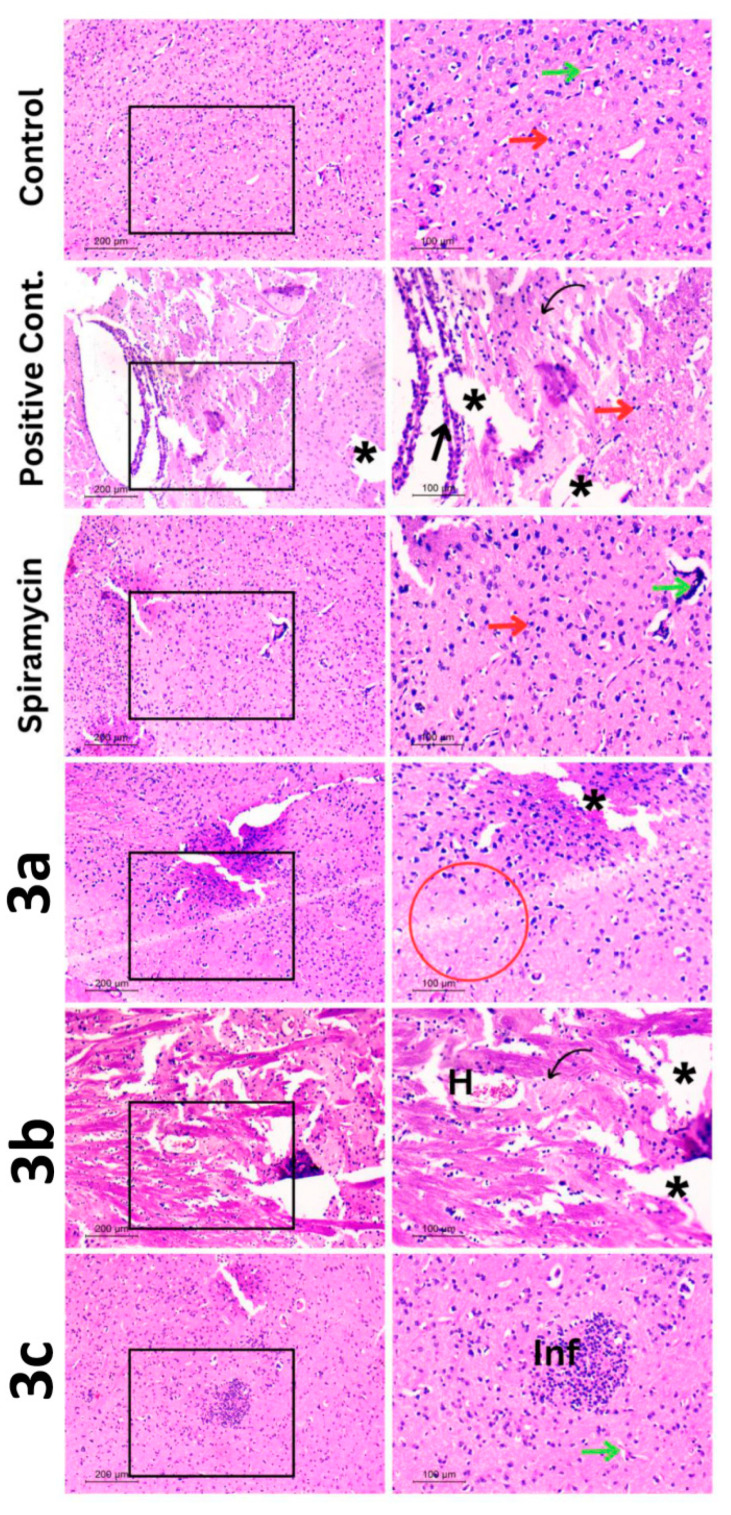
Light microscopic examination of H&E cerebral cortex-stained tissues of mice. Control group showed normal blood vessel (green arrow); normal neuron (red arrow). Positive control group showed necrotic gaps (black stars); lymphocytes (black arrow); fibrosis (bent black arrow); small neurons (red arrow). Spiramycin treated group showed normal neurons (red arrow); dilated blood vessel (green arrow). Treated group **3a** showed necrotic gap (black star); decrease neurons number (red circle). Treated group **3b** showed hemorrhagic blood vessel (H); fibrosis (black bent arrow); many necrotic gaps (black stars). Treated group **3c** showed normal neuron (green arrow) some signs of inflammation (Inf). Magnification ×(200, 100).

The control group revealed a normal neural architecture with the cell density characteristics of healthy tissue. In tissue sections, pink cells with purple cytoplasm and nuclei were seen, with no evident chromatin organization changes. Moreover, vascularization is also observed. The positive control group had localized gliosis cells, smaller neurons, and a higher eosinophilic density. Additionally, the infected mice’s brain tissues had inflammatory lesions primarily distinguished by lymphocyte infiltration into the meninges of the brain. Numerous necrotic spaces clearly disrupt the fibrosis of the neural tissue. Spiramycin, as an antibiotic drug, can overcome inflammatory changes in the brain that were observed during the early stages of infection. In summary, normal neurons are preserved while the damage stated above is minimized and gliosis is eliminated. The number of normal neurons present in the cerebral cortex and the small blood vessel dilating effects demonstrate the importance of therapy against toxoplasmosis infection, which was supported by the earlier findings. Depending on the product used (**3a-3b-3c**), the amount of tissue damage caused by the infection varied. Even though the necrotic gaps in **3a** and **3c** were less noticeable compared to **3b** or the positive control, the necrosis effect was still clearly seen in **3a** by the substantial difference in neuron density. The case of **3b** had the worst treatment outcomes, which are almost identical to the necrosis and apoptosis symptoms seen in the positive control group. A dilated blood vessel with hemorrhaging and fibrosis was also seen in this group. Additionally, compared to **3a**, **3c** had a more notable recovery in the neural tissue, but it still exhibited inflammation. The more normal neurons that are dispersed across the sectors, the more **3c** can be selective while having a few necrotic consequences (Figure 12).

##### Liver Tissue

The normal cells in the control group had rounded vesicular nuclei, polyhedral forms, vacuolated acidophilic cytoplasm, and cords extending from the central vein. Severe portal trabecular inflammation and many apoptotic signals were seen in the positive control group. The hepatic portal vein was enlarged and congested, with edema symptoms indicating an increase in hydrostatic pressure. Additionally, hepatocyte degeneration was also visible between the congested sinusoids.

In contrast to the preceding features, spiramycin produced the best effects in hepatic tissue. The central vein and hepatocytes in the liver tissue were both normal, with the exception of a small number of ballooned cells. Group **3a** of the three treatment modalities showed significantly more normal hepatocytes than the other groups. The blood sinusoids became clogged over time. Group **3b** demonstrated precisely the same attributes as the positive control, demonstrating its placebo effect. The inflamed hepatic tissue appeared with strong symptoms of edema, along with dilated, congested central veins. Fewer normal hepatocytes are located within congested blood sinusoids.

**Figure 13:**
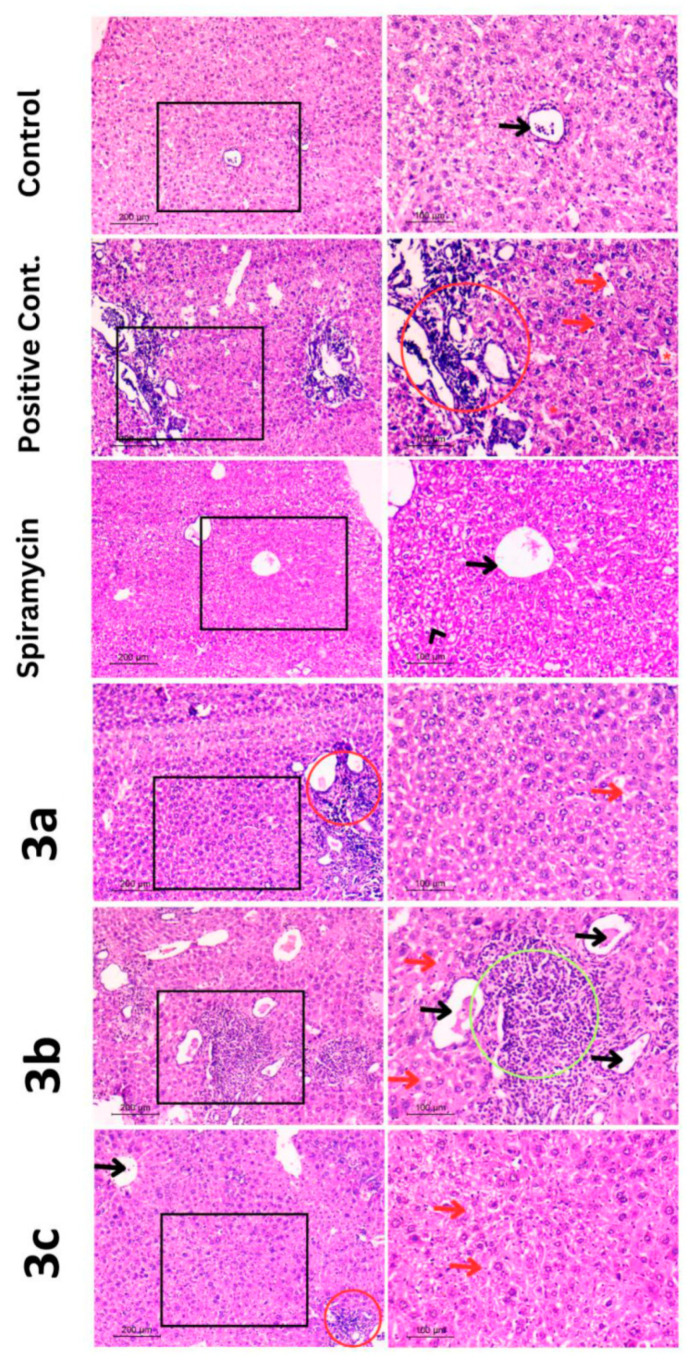
Light microscopic examination of H&E liver stained tissues of mice. Control group showed normal central vein (black arrow) with surrounding normal hepatocytes. Positive control group showed congested blood sinusoids (red arrows); degenerated hepatocytes (red stars); inflammation and congestion of portal triad (red circle). Spiramycin treated group showed normal central vein (black arrow) with few ballooned hepatocytes (black head arrow). Treated group **3a** showed congested blood sinusoids (red arrow); inflammation (red circle). Treated group **3b** showed many congested and dilated central veins (black arrows); congested blood sinusoids (red arrow); edema (green circle). Treated group **3c** showed normal central vein (black arrow); degeneration (red circle) showed congested blood sinusoids (red arrow). Magnification ×(200, 100).

Although the **3c** group had a more normal central vein than **3b** had, as well as numerous hepatocytes that were distributed normally with a normal nucleus, it nevertheless had little tissue deterioration and little swelling of the blood sinusoids (Figure 13).

##### Lung Tissue

**Figure 14:**
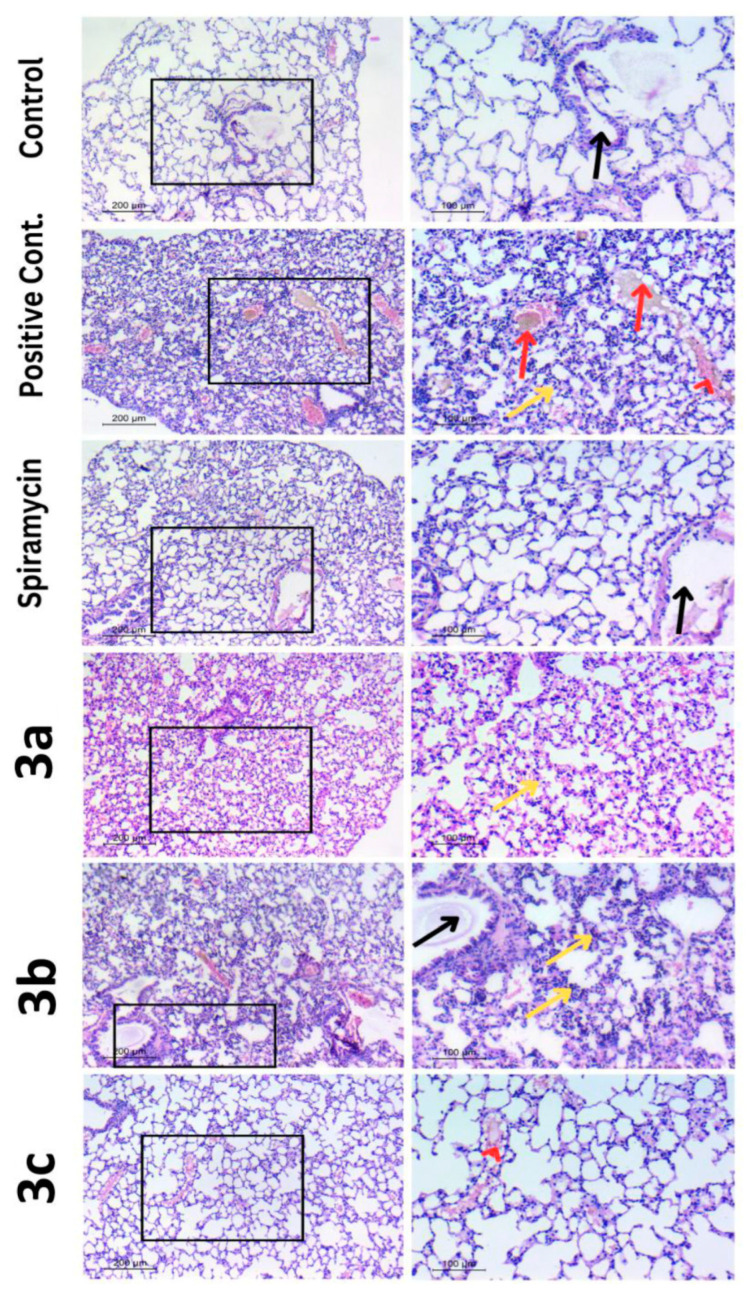
Light microscopic examination of H&E lung stained tissues of mice. Control group showed normal terminal bronchiole (black arrow) with surrounding normal Alveoli. Positive control group showed BALT (red arrows); congestion (red hard arrow); thickened epithelium (yellow arrow). Spiramycin treated group showed terminal bronchiole (black arrow) surrounded by many functional alveoli. Treated group **3a** showed thickened inflamed epithelium (yellow arrow). Treated group **3b** showed many thickened inflamed epithelia (yellow arrow); thickened wall terminal bronchiole (black arrow). Treated group **3c** showed normal alveoli diffused with some congestion (red head arrow). Magnification ×(200, 100).

In the context of the lung’s functional component (the alveolus), the results would be discussed. In the control group, the alveolar epithelium is composed of cuboidal type-2 pneumocytes and flattened squamous pulmonary type-1 cells, leaving dilated normal alveoli. The typical structures were also shown by the terminal bronchioles. The abundance of non-functional alveoli is reflected in the prominence of the bronchus-associated lymphoid tissue (BALT) in this group. Additionally, the thickness of the epithelial lung cells increased, contributing to a reduction in the efficiency of breathing by raising the physiological dead space. Spiramycin, in contrast to the preceding description, has effective alveoli and thin epithelial tissue, allowing it to effectively treat the toxoplasmosis damage symptoms. Additionally, the design of terminal bronchioles is normal, supporting the respiratory process as it applies to gas exchange. Except for a small amount of congestion, these characteristics matched the way **3c** was processed. Alveoli shrink in size in direct proportion to pulmonary epithelial thickness. Those characteristics were clearly present in **3a**; however, **3b** had the same characteristics with a different ratio, as **3b** was conjugated with the more problematic qualities, as the terminal bronchioles had a thicker wall with internal congestion (Figure 14).

## 3. Materials and Methods

### 3.1. General

Melting points were determined on a MEL-TEMP II apparatus, USA, New York and were uncorrected. ^1^H NMR spectra were determined in DMSO-*d_6_* with a Joel spectrometer, Japan, Tokyo at 500 MHz. The ^13^C NMR spectra were recorded with a Joel spectrometer at 125.7 MHz. The chemical shifts are expressed in the δ scale (Figures S1–S9; Supplementary data). FTIR spectra were recorded with a Perkin Elmer infrared spectrophotometer, USA, New York, and potassium bromide pellets and frequencies were reported in cm^−1^ (Figures S10–S12; Supplementary data). CHN analyses were carried out using a PerkinElmer 2400 Elemental Analyzer, USA, New York. All chemicals were purchased from Aldrich and used as is without further purifications.

### 3.2. General Method for the Preparation of the Hydrazones

A mixture of the indole-3-carbaldehyde (**1**) (1 g, 6.89 mmol) and the molar equivalent appropriate acid hydrazide (**2a**–**c**) (6.93 mmol) (nicotinic acid hydrazide, isonicotinic acid hydrazide, and benzoic acid hydrazide) was refluxed for 4 h in an absolute ethanol (15 mL). The formed precipitate was filtered while hot and washed with hot ethanol. The condensation reaction afforded the corresponding hydrazones (**3a**–**c**) in good yields. The products were recrystallized from DMF to give yellowish white crystals of the target compounds.

***N*’-((1*H*-indol-3-yl)methylene)nicotinohydrazide 3a:**

Yield: 61%; mp 250 °C (Decomposition). Anal. Calc. C_15_H_14_N_4_O_2_: C, 63.82; H, 5.00; N, 19.85%. Found: C, 63.65; H, 4.94; N, 19.74%. FTIR (KBr, in cm^−1^): 3249 (NH), 3042 (CH alkane), 1642 (CONH), 1574 (C=N), 1548 (C=C). ^1^HNMR (500 MHz, DMSO-*d_6_*): δ = 6.90–6.93 (m, 0.3H(H6, H5)), 7.11–7.23 (m, 1.7H (H6, H5)), 7.36 (d, 0.15H(H11), *J* = 8.1 Hz), 7.42 (d, 0.85H (H11), *J* = 8.0 Hz), 7.54–7.51 (m, 1H (H12)), 7.62 (d, 0.15H (H13), *J* = 7.9 Hz), 7.76 (d, 0.15H (H2), *J* = 2.7 Hz), 7.83 (d, 0.85H (H2), *J* = 2.7 Hz), 8.16–8.25 (m, 2H(H4, H7)), 8.58 (s, 1H(H8)), 8.71–8.75 (m, 1H (H13)), 8.97 (d, 0.15H (H10), *J* = 2.1 Hz), 9.05 (d, 0.85H (H10), *J* = 2.1 Hz), 11.53 (s, 0.15H, NH), 11.61 (s, 0.85H, NH), 11.63 (s, 0.15H, NH). ^13^C NMR: δ = 112.08, 112.40, 121.02, 122.54, 123.22, 124.10, 124.58, 130.27, 131.27, 135.83, 137.58, 146.09, 149.02, 152.45, 161.03.

***N*’-((1*H*-indol-3-yl)methylene)isonicotinohydrazide 3b:**

Yield: 66%; mp 240–244 °C, Rf 0.53 (1:2.5 EtOAc–petroleum ether). Anal. Calc. C_15_H_14_N_4_O_2_: C, 63.82; H, 5.00; N, 19.85%. Found: C, 63.67; H, 4.95; N, 19.76%. FTIR (KBr, in cm^−1^): 3165 (NH), 3047 (CH alkane), 1648 (CONH), 1590 (C=N), 1550 (C=C). ^1^HNMR (500 MHz, DMSO-*d_6_*): δ = 6.90–7.13 (m, 0.3H(H6, H5)), 7.17–7.20 (m, 1.7H(H6, H5)), 7.36 (d, 0.15H(H7), *J* = 8.0 Hz), 7.42 (d, 0.85H (H7), *J* = 8.0 Hz), 7.55 (d, 0.15H (H4), *J* = 7.9 Hz), 7.67 (d, 0.30H (H11, H12), *J* = 7.9 Hz), 7.76 (s, 0.15H(H2)), 7.81–7.83 (m, 1.70H (H11, H12)), 7.86 (s, 0.85H (H2)), 8.26 (s, 0.15H (H8)), 8.31–8.27 (m, 0.85H(H4)), 8.62 (s, 0.85H (H8)), 8.77–8.73 (m, 2H(H10, H13)), 11.54 (s, 0.15H, NH), 11.64 (s, 0.85H, NH), 11.76 (s, 1H, NH). ^13^C NMR: d = 112.04, 112.43, 121.10, 129.27, 124.85, 122.55, 122.06, 122.05, 131.51, 137.61, 141.61, 146.71, 150.78, 150.78, 161.44.

***N*’-((1*H*-indol-3-yl)methylene)benzohydrazide 3c:**

Yield: 81%; mp 234–236 °C, Rf 0.53 (1:2.5 EtOAc–petroleum ether). Anal. Calc. C_16_H_13_N_3_O: C, 72.99; H, 4.98; N, 15.96%. Found: C, 72.78; H, 4.93; N, 15.87%. FTIR (KBr, in cm^−1^): 3364 (NH), 1640 (CON), 1578 (C=N), 1549 (C=C). ^1^HNMR (500 MHz, DMSO-*d_6_*): δ = 7.11–7.20 (m, 2.5H), 7.59- 7.36 (m, 3.5H), 7.79 (s, 0.9H (H2)), 7.89 (d, 1.80H(H10, H14)) *J* = 7.2 Hz), 8.31–8.27 (m, 1H), 8.58 (s, 0.75H(H8)), 8.87 (s, 0.25H(H8)), 11.39 (s, 0.1H, NH), 11.51 (s, 0.9H, NH), 11.57 (s, 0.8H, NH), 11.66 (s, 0.2H, NH). ^13^C NMR: d = 112.25, 112.34, 120.91, 122.57, 123.16, 130.87, 128.00, 128.93, 128.93, 134.59, 137.56, 145.47, 155.64, 163.04.

### 3.3. X-ray Structure Determinations

Crystals **3b** and **3c** were immersed in cryo-oil, mounted in a loop, and measured at a temperature of 120 K. The X-ray diffraction data were collected on a Rigaku Oxford Diffraction XtaLAB Synergy R diffractometer using Cu Kα radiation. The *CrysAlisPro* [28] software package was used for cell refinement and data reduction. A multi-scan absorption correction (*CrysAlisPro* [28]) was applied to the intensities before the structure solution. The structures were solved by the intrinsic phasing (*SHELXT* [29]) method. Structural refinements were carried out using *SHELXL* [30] software with *SHELXLE* [31] graphical user interface. The NH and H_2_O hydrogen atoms were located from the difference Fourier map and refined isotropically. All other hydrogen atoms were positioned geometrically and constrained to ride on their parent atoms, with C-H = 0.95 Å and U_iso_ = 1.2·U_eq_ (parent atom). The crystallographic details are summarized in Table 11.

### 3.4. Hirshfeld Surface Analysis

Hirshfeld calculations were performed using the Crystal Explorer 17.5 program [32,33,34].

### 3.5. DFT Calculations

All DFT calculations for the indole-hydrazones were performed using Gaussian 09 software [35,36]. All calculations were performed with the B3LYP method and 6-31G(d,p) basis sets. The geometries were optimized and vibrational spectrum calculations revealed no imaginary frequencies.

### 3.6. Biology

#### 3.6.1. Cytotoxic Effects of Studied Compounds (MTT Assay)

A total of 200 µL of Human lung fibroblast (W-I38) normal cell line (ATCC: CCL-25) suspension that contained 3000 cell/well were plated in a 96-well plate for 24 h. A total of 100 µL of different concentrations of extract in RPMI medium without fetal bovine serum was added after incubation, the plate was re-incubated for additional 24 h in CO_2_ incubator. After incubation, a volume of 20 μL of MTT solution was added to each well and plates were incubated for 3 h in a CO_2_ incubator to allow the MTT to be reacted. After incubation, the plates were centrifuged at 1650 rpm for 10 min and the medium was discarded. The formazan crystals (MTT byproduct) were re-suspended in 100 μL DMSO and reading was measured at a wavelength of 570 nm using an optima spectrophotometer for detecting the safe dose, which causes 100% cell viability.

The cytotoxicity assay of the compounds (**3a**, **3b**, and **3c**) was expressed as IC50 and was calculated by the GraphPad Instat software (https://www.graphpad.com/scientific-software/instat/) using the % viability calculated from the serial dilutions of each plant extract.

#### 3.6.2. Mice

The study included sixty male Swiss albino mice, 6–8 weeks of age and weighing 20–25 g. Mice were maintained in the animal house, Pharos University in Alexandria. They were housed in wired cages under controlled temperature and light conditions and provided with water and regular mouse feed ad libitum.

#### 3.6.3. Parasites

*Toxoplasma gondii* avirulent strain (ME49) was obtained from the Theodor Bilharz Research Institute is located in Giza, Egypt. It was maintained by serial oral inoculation of mice every 8 weeks with 0.1 mL of brain suspension containing 20 cysts of previously infected mice [37].

#### 3.6.4. Drugs

Spiramycin was purchased from Sigma-Aldrich, Germany. Preparation, dose, and methods of administration were as previously described [38].

#### 3.6.5. Mice Infection

The brain of a previously infected mouse was homogenized in 1 mL saline (0.85% NaCl). A total of 0.1mL of brain suspension was placed on a glass slide and microscopically tissue homogenizer counted under a high-power lens (×40) [39].

#### 3.6.6. Mice Grouping

The study included six groups of mice, see Table 12. All mice were sacrificed at the end of the 10th week post infection (PI) for assessment of parasite burden in brain homogenates, morphological changes, and histopathological examination.

#### 3.6.7. Cyst Counts in Brain Homogenates

Smears were prepared from a half mL saline homogenate of one half of the brain of each mouse [41]. Air dried smears were stained with Giemsa stain and examined under an oil immersion lens [42]. Parasite burden was evaluated microscopically by counting the cysts in 10 HPFs of the brain homogenate smear of each mouse. Reduction percentage (R%) in cyst count was calculated according to the following equation:

R% = [(C − E)/C] × 100

where C = mean cyst count or size in the control group (GI), and E = mean cyst count or size in each treated group [43].

#### 3.6.8. Morphological Changes

Brain cysts from each studied group were harvested in a 2.5% glutaraldehyde solution, they were examined by scanning electron microscope (SEM) to detect morphological changes [44].

#### 3.6.9. Histopathological Examination

For the detection of pathological changes in the brains of mice infected with the Me49 Toxoplasma strain, a part of the brain tissues from all studied groups was fixed in 10 % formalin using hematoxylin and eosin stain (H&E) [45].

## 4. Conclusions

Three indole-hydrazones of nicotinic, isonicotinic, and benzoic acid hydrazides were synthesized and characterized with the aid of different experimental and theoretical methods. Structural analyses revealed the extrastability of the ***EAnti*** form in polar aprotic (DMSO) and polar protic (ethanol) solvents, as well as in solid phase. On the other hand, the ***ESyn*** form is the most stable in gas phase and in the nonpolar solvents (CCl_4_ and cyclohexane). The molecular and supramolecular structures of hydrazones **3b** and **3c** were investigated based on the X-ray crystal structural and Hirshfeld analyses. The intermolecular interactions N…H, O…H, H…C, and π…π have great importance for the crystal stability. The hydrazone **3b** (5.7234 Debye) has the highest polar character compared to the others. Moreover, the **3a**–**c** were used in the treatment of *T. gondii* infected mice and the results of our current study showed that the all **3a**–**c** significantly reduced the parasite load in brains of treated mice, particularly, **3c** had a promising therapeutic role against chronic toxoplasmosis.

## Figures and Tables

**Table 1:** Selected bond lengths [Å] and angles [°] for **3b**.

Bond Distances			
O(1)-C(6)	1.2283(12)	N(2)-N(3)	1.3963(11)
N(1)-C(3)	1.3382(14)	N(3)-C(7)	1.2883(13)
N(1)-C(2)	1.3406(14)	N(4)-C(15)	1.3567(13)
N(2)-C(6)	1.3476(13)	N(4)-C(14)	1.3823(12)
**Bond Angles**			
C(3)-N(1)-C(2)	117.12(8)	C(2)-C(1)-C(5)	118.63(9)
C(6)-N(2)-N(3)	118.60(8)	N(1)-C(2)-C(1)	123.67(9)
C(7)-N(3)-N(2)	113.43(8)	N(1)-C(3)-C(4)	123.49(9)
C(15)-N(4)-C(14)	108.78(8)	C(3)-C(4)-C(5)	118.71(9)

**Table 2:** Hydrogen bonds for **3b** [Å] and [°].

D-H…A	d(D-H)	d(H…A)	d(D…A)	<(DHA)
N2-H2…O2 ^#1^	0.896(15)	2.057(15)	2.8903(12)	154.4(14)
O2-H2B…N3 ^#2^	0.870(17)	2.283(17)	3.0927(12)	154.9(13)
O2-H2C…O1	0.897(15)	1.886(15)	2.7705(11)	168.5(15)
N4-H4…N1 ^#3^	0.913(15)	2.014(15)	2.9120(11)	167.4(13)
C4-H4A…O2 ^#1^	0.95	2.52	3.4231(13)	159

^#1^: 1 + x,y,z; ^#2^: 1 − x, 1 − y, 1 − z; ^#3^: 2 − x, −1/2 + y, 1/2 − z.

**Table 3:** Selected bond lengths [Å] and angles [°] for **3c**.

Bond Distances			
O(1)-C(7)	1.2445(12)	N(2)-C(8)	1.2884(13)
N(1)-C(7)	1.3410(12)	N(3)-C(16)	1.3591(13)
N(1)-N(2)	1.3925(11)	N(3)-C(15)	1.3817(13)
O(1)-C(7)	1.2445(12)	N(2)-C(8)	1.2884(13)
**Bond Angles**			
C(7)-N(1)-N(2)	121.52(8)	C(3)-C(2)-C(1)	120.03(10)
C(8)-N(2)-N(1)	112.43(8)	C(4)-C(3)-C(2)	120.04(10)
C(16)-N(3)-C(15)	109.02(8)	C(3)-C(4)-C(5)	120.23(10)
C(2)-C(1)-C(6)	120.16(9)	C(4)-C(5)-C(6)	119.89(10)

**Table 4:** Hydrogen bonds for **3c** [Å and °].

D-H…A	d(D-H)	d(H…A)	d(D…A)	<(DHA)
N1-H1…O1 ^#1^	0.902(15)	1.981(15)	2.8827(11)	178.3(13)
N3-H3…O1 ^#2^	0.919(16)	1.936(16)	2.8304(11)	163.9(13)

Symm. Codes: ^#1^: −x + 1/2, y + 1/2, z ^#2^: x + 1/2, y, −z + 3/2.

**Table 5:** All short distance contacts in **3c**.

Contact	Distance	Contact	Distance
H2…C12	2.649	O1…H1	1.874
H1…C7	2.715	O3…H1	1.850
H**3a**…C15	2.557	C1…C2	3.323
H**3a**…C10	2.696	C1…C7	3.353
H14…C4	2.785	H11…N2	2.541
H16…C10	2.703	H11…N1	2.568
H16…C15	2.754		

**Table 6:** All short distance contacts in **3b**.

Contact	Distance	Contact	Distance
C8…H13	2.761	N1…H4	1.921
C15…H13	2.719	N3…H2B	2.181
C5…H11	2.701	O1…H2C	1.802
C3…H4	2.689	O2…H2	1.955
O2…H4A	2.558	O2…H7	2.394

**Table 7:** Energetics (A.U.) of the studied conformers for **3b** and **3c** in different solvents.

Parameter	*EAnti*	*ESyn*	*ZAnti*	*ZSyn*
**3b (gas phase)**				
E_corr_^b^	−872.8016	−872.8040	−872.7960	−872.8006
∆E	−1.4964	0.0000	−5.0347	−2.0974
%	7.20	90.8	0.02	1.98
**3b (DMSO)**				
E_corr_^b^	−872.8220	−872.8206	−872.8169	−872.8168
∆E	0.0000	−0.8522	−3.2167	−3.2524
%	80.04	19.60	0.18	0.17
**3b (Ethanol)**				
E_corr_^b^	−872.8213	−872.8201	−872.8161	−872.8163
∆E	0.0000	−0.7425	−3.2442	−3.1348
%	77.15	22.49	0.15	0.20
**3b (CCl_4_)**				
E_corr_^b^	−872.8100	−872.8111	−872.8045	−872.8076
∆E	−0.7085	0.0000	−4.1583	−2.2119
%	26.14	72.79	0.04	1.03
**3b (Cyclohexane)**				
E_corr_^a^	−872.8090	−872.8103	−872.8035	−872.8068
∆E ^b^	−0.8066	0.0000	−4.2648	−2.1959
%	23.45	75.39	0.04	1.12
**3c (gas phase)**				
E_corr_^a^	−856.7569	−856.7588	−856.7512	−856.7557
∆E ^b^	−1.2179	0.0000	−4.7603	−1.9775
%	10.1	87.9	0.1	1.9
**3c (DMSO)**				
E_corr_^a^	−856.7760	−856.7742	−856.7710	−856.7706
∆E ^b^	0.0000	−1.1399	−3.1369	−3.3940
%	86.41	13.18	0.26	0.14
**3c (Ethanol)**				
E_corr_^a^	−856.7754	−856.7737	−856.7703	−856.7701
∆E^b^	0.0000	−1.0408	−3.1651	−3.2804
%	85.05	14.54	0.23	0.18
**3c (CCl_4_)**				
E_corr_^a^	−856.7646	−856.7652	−856.7593	−856.7620
∆E ^b^	−0.3803	0.0000	−3.7345	−2.0561
%	39.43	59.25	0.14	1.18
**3c (Cyclohexane)**				
E_corr_^a^	−856.7638	−856.7645	−856.7584	−856.7613
∆E ^b^	−0.4839	0.0000	−3.8437	−2.0415
%	34.85	63.68	0.14	1.33

^a^ E_corr_ = E_tot_ + ZPVE, ^b^ ∆E is the difference between the E_corr_ values for an isomer and the most stable one.

**Table 8:** Energetics (A.U.) of the studied conformers for **3a** in different solvents.

Parameter	*EAnti*1	*EAnti*2	*ESyn*1	*ESyn*2	*ZAnti*1	*ZAnti*2	*ZSny*1	*ZSyn*2
**Gas phase**								
E_corr_^a^	−872.8012	−872.8027	−872.8054	−872.8045	−872.7954	−872.7976	−872.8017	−872.8014
∆E ^b^	−2.5941	−1.6893	0.0000	−0.5733	−6.2611	−4.9028	−2.3093	−2.4622
%	0.77	3.90	67.90	26.00	0.03	0.01	0.75	0.64
**DMSO**								
E_corr_^a^	−872.8225	−872.8227	−872.8217	−872.8217	−872.8174	−872.8176	−872.8179	−872.8179
∆E ^b^	−0.1307	0.0000	−0.6171	−0.6174	−3.3053	−3.1926	−2.9676	−2.9727
%	29.33	37.90	17.42	14.92	0.09	0.08	0.13	0.13
**Ethanol**								
E_corr_^a^	−872.8217	−872.8220	−872.8211	−872.8211	−872.8174	−872.8169	−872.8174	−872.8174
∆E ^b^	−0.1663	0.0000	−0.5110	−0.5388	−2.8637	−3.1917	−2.8616	−2.8776
%	27.09	35.87	19.69	16.81	0.17	0.08	0.15	0.14
**CCl_4_**								
E_corr_^a^	−872.8099	−872.8109	−872.8124	−872.8117	−872.8042	−872.8059	−872.8087	−872.8085
∆E ^b^	−1.5448	−0.9240	0.0000	−0.4105	−5.1277	−4.0444	−2.3307	−2.4386
%	5.02	12.42	56.49	24.93	0.00	0.06	0.58	0.49
**Cyclohexane**								
E_corr_^a^	−872.8090	−872.8100	−872.8116	−872.8109	−872.8032	−872.8051	−872.8079	−872.8077
∆E ^b^	−1.6783	−1.0354	0.0000	−0.4343	−5.2781	−4.1308	−2.3292	−2.4422
%	4.08	10.95	58.75	25.04	0.00	0.07	0.60	0.52

^a^ E_corr_ = E_tot_ + ZPVE, ^b^ ∆E is the difference between the E_corr_ values for an isomer and the most stable one.

**Table 9:** Natural charges at different atomic sites of the studied systems.

Atom	Charge	Atom	Charge	Atom	Charge
3a		3b		3c	
C1	0.042	C1	0.042	C1	0.036
H2	0.190	H2	0.190	H2	0.189
C3	0.677	C3	0.678	C3	0.680
H4	0.403	H4	0.403	H4	0.400
C5	0.154	C5	0.154	C5	0.155
C6	−0.076	C6	−0.076	C6	−0.077
C7	−0.222	C7	−0.222	C7	−0.221
C8	−0.253	C8	−0.252	C8	−0.253
C9	−0.240	C9	−0.240	C9	−0.241
C10	−0.264	C10	−0.264	C10	−0.265
C11	0.021	C11	0.020	C11	0.016
C12	−0.178	C12	−0.177	C12	−0.175
H13	0.237	H13	0.238	H13	0.237
H14	0.241	H14	0.242	H14	0.241
H15	0.242	H15	0.242	H15	0.241
H16	0.240	H16	0.241	H16	0.240
H17	0.442	H17	0.442	H17	0.441
H18	0.253	H18	0.251	H18	0.250
N19	−0.277	N19	−0.278	N19	−0.272
N20	−0.446	N20	−0.446	N20	−0.449
N21	−0.550	N21	−0.550	N21	−0.552
O22	−0.611	O22	−0.603	O22	−0.612
C23	−0.186	C23	−0.116	C23	−0.149
C24	0.054	C24	−0.245	C24	−0.201
C25	−0.161	C25	−0.234	C25	−0.190
H26	0.243	C26	0.010	C26	−0.245
C27	−0.284	H27	0.261	H27	0.257
H28	0.265	C28	0.011	C28	−0.243
C29	0.029	H29	0.264	H29	0.261
H30	0.247	H30	0.225	C30	−0.221
H31	0.226	H31	0.228	H31	0.239
N32	−0.458	N32	−0.440	H32	0.242

**Table 10:** The effect of **3a**–**c** and spiramycin on mean cyst count (/10HPFs) and R% in brain homogenate *T. gondii* infected groups.

Groups	Cyst Count		Statistical Analysis
	Mean ± SD	Reduction%	F Test (*p* Value)
Group II	22.9 ± 1.7		138.43 (*p* < 0.01)
Group III	11.1 ± 1.4 ^a,c,d,e^	52%	
Group IV	14.4 ± 1.1 ^a,b,d,e^	37%	
Group V	15.6 ± 1.2 ^a,b,c,e^	32%	
Group VI	11.6 ± 1.3 ^a,b,c,d^	49%	

SD: standard deviation, F: F-test (ANOVA) and *p* ≤ 0.05 (statistically significant). ^a^ Significant with group II, ^b^ Significant with group III, ^c^ Significant with group IV, ^d^ Significant with group V, ^e^ Significant with group VI.

**Table 11:** Crystal data of **3b** and **3c**.

	3b	3c
CCDC	2,279,371	2,279,372
empirical formula	C_15_H_14_N_4_O_2_	C_16_H_13_N_3_O
fw	282.30	263.29
temp (K)	120(2)	120(2)
*λ*(Å)	1.54184	1.54184
cryst syst	Monoclinic	Orthorhombic
space group	*P2* *_1_* */c*	*Pbca*
*a* (Å)	7.04998(8)	17.42273(12)
*b* (Å)	25.0269(3)	7.69675(5)
*c* (Å)	7.88162(9)	19.92797(14)
β (deg)	104.1652(11)	90
*V* (Å^3^)	1348.35(3)	2672.31(3)
Z	4	8
*ρ*_calc_ (mg/m^3^)	1.391	1.309
*μ*(Mo K*α*) (mm^−1^)	0.788	0.680
No. reflns.	12,347	60,334
Unique reflns.	2851	2886
Completeness to θ = 67.684°	99.7%	100%
GOOF (*F*^2^)	1.061	1.051
*R*_int_	0.0141	0.0389
*R*_1_^a^ (*I* ≥ 2σ)	0.0321	0.0317
*wR*_2_^b^ (*I* ≥ 2σ)	0.0859	0.0834

^a^ *R*_1_ = Σ||F_o_| – |F_c_||/Σ|F_o_|. ^b^ *wR*_2_ = {Σ[w(F_o_^2^ – F_c_^2^)^2^]/Σ[w(F_o_^2^)^2^]}^1/2^.

**Table 12:** Mice grouping details.

Group	No.	Name	Characteristics
Group I	10	Negative control	non-infected, non-treated
Group II	10	Positive control	Infected, non-treated
Group III	10	Spiramycin	Infected and treated with Spiramycin 100 mg/kg once daily for 10 d after 6 w PI [40]
Group IV	10	**3a**	and treated with **3a** 100 mg/kg once daily for 14 d after 8 w PI
Group V	10	**3b**	Infected and treated with **3b** 100 mg/kg once daily for 14 d after 8 w PI
Group VI	10	**3c**	Infected and treated with **3c** 100 mg/kg once daily for 14 d after 8 w PI

## Data Availability

Data are contained within the article or supplementary material.

## Supplementary Materials

The following supporting information can be downloaded at: https://www.mdpi.com/article/10.3390/ijms241713251/s1.
