# Supplementary material for: Synthesis, X-ray Structure, Hirshfeld, DFT Conformational, Cytotoxic, and Anti-Toxoplasma Studies of New Indole-Hydrazone Derivatives
Source: Int J Mol Sci. 2023 Aug 26;24(17):13251. doi: 10.3390/ijms241713251 (PMC10487720; doi:10.3390/ijms241713251)
Supplement: Supplementary file 1 [file ijms-24-13251-s001.zip › ijms-2530565-supplementary.pdf]

SUPPORTING MATERIALS

Table S1 Energetics (A.U.) of the studied conformers for **3b** in gas phase.

Parameter	*EAnti*	*ESyn*	*ZAnti*	*ZSny*
E	-873.0491	-873.0517	-873.0441	-873.0491
ZPVE[a]	0.2475	0.2477	0.2481	0.2485
E_{corr}[b]	-872.8016	-872.8040	-872.7960	-872.8006
ΔE ($kcal.mol^{-1}$) [c]	-1.4964	0.0000	-5.0347	-2.0974
H	-872.7846	-872.7871	-872.7792	-872.7840
G	-872.8477	-872.8501	-872.8421	-872.8464
S ($cal.mol^{-1}K^{-1}$)	132.7640	132.5730	132.3330	131.4030
G(kcal/mol)	-547720.2188	-547721.7166	-547716.6916	-547719.4043
K	12.6600		4995.9	50.3000
%	7.2000	90.8	0.018	1.9820

[a]Zero point energy correction [b]E+ZPVE [c] E_{form}-E_{ESyn}

Table S2 Energetics (A.U.) of the studied conformers for **3b** in DMSO.

Parameter	*EAnti*	*ESyn*	*ZAnti*	*ZSny*
E	-873.0699	-873.0685	-873.0654	-873.0654
ZPVE[a]	0.2479	0.2479	0.2485	0.2486
E_{corr}[b]	-872.8220	-872.8206	-872.8169	-872.8168
ΔE ($kcal.mol^{-1}$) [c]	0.0000	-0.8522	-3.2167	-3.2524
H	-872.8051	-872.8038	-872.8002	-872.8002
G	-872.8681	-872.8668	-872.8624	-872.8624
S ($cal.mol^{-1}K^{-1}$)	132.6790	132.6610	130.9160	130.8640
G(kcal/mol)	-547733.0444	-547732.2143	-547729.4614	-547729.4200
K	1	4.083	433.808	465.352
%	80.04	19.60	0.18	0.17

[a]Zero point energy correction [b]E+ZPVE [c] E_{form}-E_{ESyn}

Table S3 Energetics (A.U.) of the studied conformers for **3b** in ethanol.

Parameter	*EAnti*	*ESyn*	*ZAnti*	*ZSny*
E	-873.0691	-873.0680	-873.0646	-873.0648
ZPVE[a]	0.2478	0.2479	0.2485	0.2486
E_{corr}[b]	-872.8213	-872.8201	-872.8161	-872.8163
ΔE (kcal.mol^{-1}) [c]	0.0000	-0.7425	-3.2442	-3.1348
H	-872.8044	-872.8032	-872.7995	-872.7996
G	-872.8674	-872.8663	-872.8616	-872.8618
S (cal.mol^{-1}K^{-1})	132.7430	132.7000	130.7730	130.9170
G(kcal/mol)	-547732.6071	-547731.8798	-547728.9412	-547729.0980
K	1.0000	3.4301	499.1898	382.6461
%	77.15	22.49	0.15	0.20

[a]Zero point energy correction [b]E+ZPVE [c] E_{form}-E_{ESyn}

Table S4 Energetics (A.U.) of the studied conformers for **3b** in CCl_4.

Parameter	*EAnti*	*ESyn*	*ZAnti*	*ZSny*
E	-873.0576	-873.0589	-873.0528	-873.0562
ZPVE[a]	0.2476	0.2478	0.2483	0.2486
E_{corr}[b]	-872.8100	-872.8111	-872.8045	-872.8076
ΔE (kcal.mol^{-1}) [c]	-0.7085	0.0000	-4.1583	-2.2119
H	-872.7930	-872.7942	-872.7878	-872.7909
G	-872.8560	-872.8570	-872.8499	-872.8530
S (cal.mol^{-1}K^{-1})	132.6030	132.0730	130.6530	130.5800
G(kcal/mol)	-547725.4497	-547726.0540	-547721.5930	-547723.5408
K	2.7847		1920.7003	70.7626
%	26.14	72.79	0.04	1.03

[a]Zero point energy correction [b]E+ZPVE [c] E_{form}-E_{ESyn}

Table S5 Energetics (A.U.) of the studied conformers for **3b** in cyclohexane.

Parameter	*EAnti*	*ESyn*	*ZAnti*	*ZSny*
E	-873.0567	-873.0581	-873.0519	-873.0554
ZPVE[a]	0.2476	0.2478	0.2483	0.2486
E_{corr}[b]	-872.8090	-872.8103	-872.8035	-872.8068
ΔE (kcal.mol^{-1}) [c]	-0.8066	0.0000	-4.2648	-2.1959
H	-872.7921	-872.7935	-872.7869	-872.7902
G	-872.8551	-872.8562	-872.8490	-872.8523
S (cal.mol^{-1}K^{-1})	132.6270	132.0440	130.8220	130.6080
G(kcal/mol)	-547724.8718	-547725.5608	-547721.0452	-547723.0790
K	3.2147		2106.8936	67.0981
%	23.45	75.39	0.04	1.12

[a]Zero point energy correction [b]E+ZPVE [c] E_{form}-E_{ESyn}

Table S6 Energetics (A.U.) of the studied conformers for **3c** in gas phase.

Parameter	*EAnti*	*ESyn*	*ZAnti*	*ZSny*
E	-857.0161	-857.0183	-857.0112	-857.0160
ZPVE[a]	0.2593	0.2595	0.2599	0.2603
E_{corr}[b]	-856.7569	-856.7588	-856.7512	-856.7557
ΔE (kcal.mol^{-1}) [c]	-1.2179	0.0000	-4.7603	-1.9775
H	-856.7398	-856.7418	-856.7343	-856.7389
G	-856.8029	-856.8049	-856.7973	-856.8013
S (cal.mol^{-1}K^{-1})	132.9250	132.9420	132.4420	131.5140
G(kcal/mol)	-537651.9707	-537653.2426	-537648.4227	-537650.9811
K	8.6300		3528.9000	46.1900
%	10.1	87.9	0.1	1.9

[a]Zero point energy correction [b]E+ZPVE [c] E_{form}-E_{ESyn}

Table S7 Energetics (A.U.) of the studied conformers for **3c** in DMSO.

Parameter	*EAnti*	*ESyn*	*ZAnti*	*ZSny*
E	-857.0357	-857.0339	-857.0314	-857.0310
ZPVE[a]	0.2596	0.2596	0.2603	0.2603
E_{corr}[b]	-856.7760	-856.7742	-856.7710	-856.7706
ΔE ($kcal.mol^{-1}$) [c]	0.0000	-1.1399	-3.1369	-3.3940
H	-856.7590	-856.7572	-856.7543	-856.7539
G	-856.8221	-856.8204	-856.8167	-856.8161
S ($cal.mol^{-1}K^{-1}$)	132.8200	132.8360	131.2960	130.7420
G(kcal/mol)	-537664.0239	-537662.9144	-537660.5977	-537660.2513
K	1.0000	6.5553	332.5294	598.1144
%	86.41	13.18	0.26	0.14

[a]Zero point energy correction [b]E+ZPVE [c] E_{form}-E_{ESyn}

Table S8 Energetics (A.U.) of the studied conformers for **3c** in ethanol.

Parameter	*EAnti*	*ESyn*	*ZAnti*	*ZSny*
E	-857.0349	-857.0333	-857.0306	-857.0305
ZPVE[a]	0.2596	0.2596	0.2603	0.2603
E_{corr}[b]	-856.7754	-856.7737	-856.7703	-856.7701
ΔE ($kcal.mol^{-1}$) [c]	0.0000	-1.0408	-3.1651	-3.2804
H	-856.7583	-856.7567	-856.7535	-856.7534
G	-856.8215	-856.8198	-856.8159	-856.8157
S ($cal.mol^{-1}K^{-1}$)	132.8970	132.7940	131.2440	131.0740
G(kcal/mol)	-537663.6085	-537662.5662	-537660.1208	-537659.9633
K	1	5.8502	369.0571	481.9743
%	85.05	14.54	0.23	0.18

[a]Zero point energy correction [b]E+ZPVE [c] E_{form}-E_{ESyn}

Table S9 Energetics (A.U.) of the studied conformers for **3c** in CCl_4.

Parameter	*EAnti*	*ESyn*	*ZAnti*	*ZSny*
E	-857.0241	-857.0248	-857.0194	-857.0223
ZPVE[a]	0.2594	0.2596	0.2601	0.2604
E_{corr}[b]	-856.7646	-856.7652	-856.7593	-856.7620
ΔE ($kcal.mol^{-1}$) [c]	-0.3803	0.0000	-3.7345	-2.0561
H	-856.7476	-856.7483	-856.7425	-856.7452
G	-856.8106	-856.8110	-856.8053	-856.8073
S ($cal.mol^{-1}K^{-1}$)	132.7240	132.0730	132.2740	130.7420
G(kcal/mol)	-537656.8113	-537657.0516	-537653.4660	-537654.7405
K	1.5028		435.6582	50.2436
%	39.43	59.25	0.14	1.18

[a]Zero point energy correction [b]E+ZPVE [c] E_{form}-E_{ESyn}

Table S10 Energetics (A.U.) of the studied conformers for **3c** in cyclohexane.

Parameter	*EAnti*	*ESyn*	*ZAnti*	*ZSny*
E	-857.0232	-857.0241	-857.0185	-857.0216
ZPVE[a]	0.2594	0.2596	0.2600	0.2604
E_{corr}[b]	-856.7638	-856.7645	-856.7584	-856.7613
ΔE ($kcal.mol^{-1}$) [c]	-0.4839	0.0000	-3.8437	-2.0415
H	-856.7467	-856.7476	-856.7416	-856.7445
G	-856.8098	-856.8103	-856.8045	-856.8067
S ($cal.mol^{-1}K^{-1}$)	132.7290	132.1160	132.5750	130.8410
G(kcal/mol)	-537656.2641	-537656.6199	-537652.9935	-537654.3383
K	1.8276		466.8396	47.7939
%	34.85	63.68	0.14	1.33

[a]Zero point energy correction [b]E+ZPVE [c] E_{form}-E_{ESyn}

Table S11 Energetics (A.U.) of the studied conformers for **3a** in gas phase.

**Parameter**	***EAnti1***	***EAnti2***	***ESyn1***	***ESyn2***	***ZAnti1***	***ZAnti2***	***ZSny1***	***ZSyn2***
E	-873.0487	-873.0502	-873.0532	-873.0522	-873.0435	-873.0458	-873.0502	-873.0500
ZPVE[a]	0.2474	0.2475	0.2478	0.2477	0.2481	0.2482	0.2485	0.2485
E_{corr}[b]	-872.8012	-872.8027	-872.8054	-872.8045	-872.7954	-872.7976	-872.8017	-872.8014
ΔE (kcal.mol^{-1}) [c]	-2.5941	-1.6893	0.0000	-0.5733	-6.2611	-4.9028	-2.3093	-2.4622
H	-872.7842	-872.7857	-872.7885	-872.7875	-872.7786	-872.7808	-872.7850	-872.7848
G	-872.8472	-872.8487	-872.8514	-872.8505	-872.8415	-872.8435	-872.8472	-872.8470
S (cal.mol^{-1}K^{-1})	132.5900	132.6830	132.5600	132.6090	132.3240	131.9540	130.9030	131.1090
G (kcal.mol^{-1})	-547719.925	-547720.864	-547722.574	-547722.007	-547716.313	-547717.582	-547719.909	-547719.813
K	89.1300	18.1400		2.6200	40605.3000	4728.2000	91.5300	107.7000
%	0.77	3.90	67.90	26.00	0.03	0.01	0.75	0.64

[a]Zero point energy correction [b]E+ZPVE [c] E_{form}-E_{ESyn}

Table S12 Energetics (A.U.) of the studied conformers for **3a** in DMSO.

**parameter**	**EAnti1**	**EAnti2**	**ESyn1**	**ESyn2**	**ZAnti1**	**ZAnti2**	**ZSny1**	**ZSyn2**
E	-873.0703	-873.0706	-873.0696	-873.0696	-873.0659	-873.0662	-873.0666	-873.0666
ZPVE[a]	0.2479	0.2479	0.2479	0.2479	0.2485	0.2486	0.2486	0.2486
E_{corr}[b]	-872.8225	-872.8227	-872.8217	-872.8217	-872.8174	-872.8176	-872.8179	-872.8179
ΔE (kcal.mol^{-1}) [c]	-0.1307	0.0000	-0.6171	-0.6174	-3.3053	-3.1926	-2.9676	-2.9727
H	-872.8055	-872.8057	-872.8048	-872.8048	-872.8007	-872.8009	-872.8013	-872.8013
G	-872.8684	-872.8687	-872.8679	-872.8678	-872.8630	-872.8629	-872.8634	-872.8633
S (cal.mol^{-1}K^{-1})	S	132.3680	132.4400	132.9040	132.5620	130.9650	130.3780	130.6000
G (kcal.mol^{-1})	-547733.235	-547733.386	-547732.927	-547732.835	-547729.795	-547729.759	-547730.046	-547730.014
K	1.2921	1.0000	2.1759	2.5413	439.8481	467.3363	287.4440	303.1426
%	29.33	37.90	17.42	14.92	0.09	0.08	0.13	0.13

[a]Zero point energy correction [b]E+ZPVE [c] E_{form}-E_{ESyn}

Table S13 Energetics (A.U.) of the studied conformers for **3a** in ethanol.

**parameter**	***EAnti1***	***EAnti2***	***ESyn1***	***ESyn2***	***ZAnti1***	***ZAnti2***	***ZSny1***	***ZSyn2***
E	-873.0696	-873.0698	-873.0690	-873.0690	-873.0659	-873.0655	-873.0660	-873.0660
ZPVE[a]	0.2479	0.2479	0.2479	0.2479	0.2485	0.2486	0.2486	0.2486
E_{corr}[b]	-872.8217	-872.8220	-872.8211	-872.8211	-872.8174	-872.8169	-872.8174	-872.8174
ΔE (kcal.mol^{-1}) [c]	-0.1663	0.0000	-0.5110	-0.5388	-2.8637	-3.1917	-2.8616	-2.8776
H	-872.8048	-872.8050	-872.8043	-872.8042	-872.8007	-872.8002	-872.8008	-872.8007
G	-872.8677	-872.8680	-872.8674	-872.8673	-872.8630	-872.8622	-872.8628	-872.8627
S (cal.mol^{-1}K^{-1})	132.4540	132.4460	132.9010	132.6530	130.9650	130.4240	130.6280	130.4740
G (kcal.mol^{-1})	-547732.778	-547732.944	-547732.590	-547732.497	-547729.795	-547729.329	-547729.717	-547729.661
K	1.3241	1.0000	1.8218	2.1346	208.0385	457.9878	237.1124	260.9293
%	27.09	35.87	19.69	16.81	0.17	0.08	0.15	0.14

[a]Zero point energy correction [b]E+ZPVE [c] E_{form}-E_{ESyn}

Table S14 Energetics (A.U.) of the studied conformers for **3a** in CCl_4.

Parameter	*EAnti1*	*EAnti2*	*ESyn1*	*ESyn2*	*ZAnti1*	*ZAnti2*	*ZSny1*	*ZSyn2*
E	-873.0575	-873.0586	-873.0602	-873.0596	-873.0526	-873.0543	-873.0573	-873.0571
ZPVE[a]	0.2476	0.2477	0.2478	0.2478	0.2484	0.2484	0.2486	0.2486
E_{corr}[b]	-872.8099	-872.8109	-872.8124	-872.8117	-872.8042	-872.8059	-872.8087	-872.8085
ΔE (kcal.mol^{-1}) [c]	-1.5448	-0.9240	0.0000	-0.4105	-5.1277	-4.0444	-2.3307	-2.4386
H	-872.7929	-872.7940	-872.7955	-872.7949	-872.7875	-872.7892	-872.7920	-872.7919
G	-872.8560	-872.8569	-872.8583	-872.8575	-872.8495	-872.8519	-872.8540	-872.8538
S (cal.mol^{-1}K^{-1})	132.7720	132.4010	132.1420	131.8910	130.3620	131.8060	130.4060	130.4530
G (kcal.mol^{-1})	-547725.447	-547725.982	-547726.875	-547726.393	-547721.342	-547722.828	-547724.173	-547724.078
K	11.2519	4.5468	1.0000	2.2656	11825.0128	952.9824	97.4603	114.5599
%	5.02	12.42	56.49	24.93	0.00	0.06	0.58	0.49

[a]Zero point energy correction [b]E+ZPVE [c] E_{form}-E_{ESyn}

Table S15 Energetics (A.U.) of the studied conformers for **3a** in cyclohexane.

parameter	*EAnti1*	*EAnti2*	*ESyn1*	*ESyn2*	*ZAnti1*	*ZAnti2*	*ZSny1*	*ZSyn2*
E	-873.0565	-873.0577	-873.0595	-873.0588	-873.0516	-873.0534	-873.0565	-873.0563
ZPVE[a]	0.2476	0.2477	0.2478	0.2478	0.2483	0.2483	0.2486	0.2486
E_{corr}[b]	-872.8090	-872.8100	-872.8116	-872.8109	-872.8032	-872.8051	-872.8079	-872.8077
ΔE (kcal.mol^{-1}) [c]	-1.6783	-1.0354	0.0000	-0.4343	-5.2781	-4.1308	-2.3292	-2.4422
H	-872.7920	-872.7931	-872.7948	-872.7941	-872.7865	-872.7883	-872.7913	-872.7911
G	-872.8550	-872.8560	-872.8576	-872.8568	-872.8485	-872.8512	-872.8532	-872.8531
S (cal.mol^{-1}K^{-1})	132.7540	132.4310	132.1630	131.9240	130.4580	132.2660	130.4120	130.5060
G (kcal.mol^{-1})	-547724.833	-547725.415	-547726.407	-547725.903	-547720.741	-547722.393	-547723.701	-547723.615
K	14.3852	5.3674	1.0000	2.3465	14799.8377	899.7953	97.9799	113.4686
%	4.08	10.95	58.75	25.04	0.00	0.07	0.60	0.52

[a]Zero point energy correction [b]E+ZPVE [c] E_{form}-E_{ESyn}

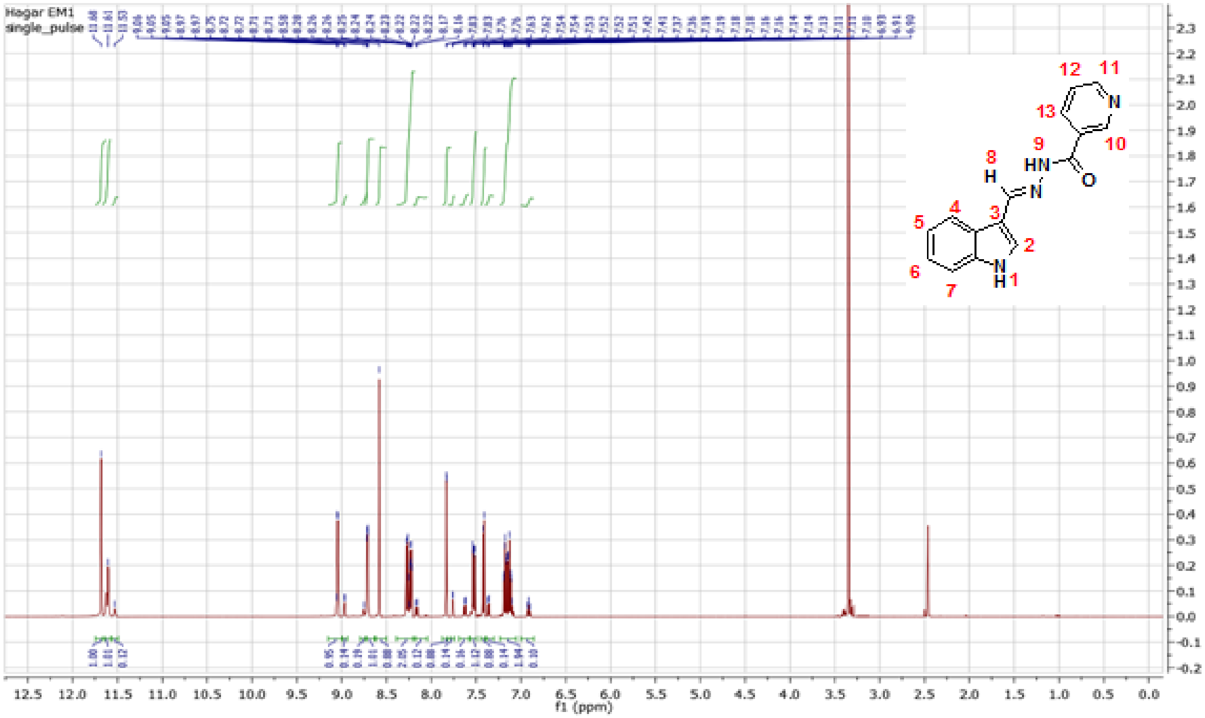

Figure S1 ^{1}H NMR spectrum of **3a**.

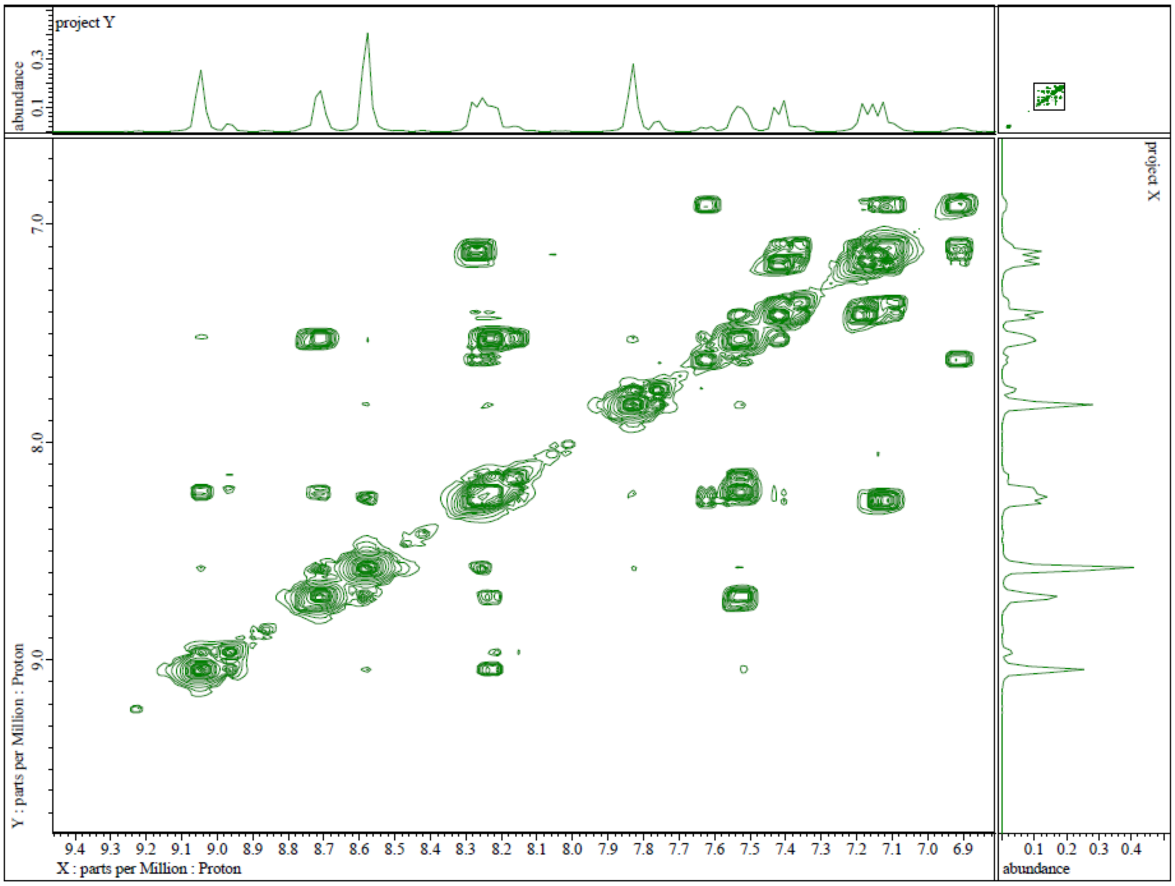

Figure S2 COSY spectrum of **3a**.

Figure S3 ^{13}C NMR spectrum of **3a**.

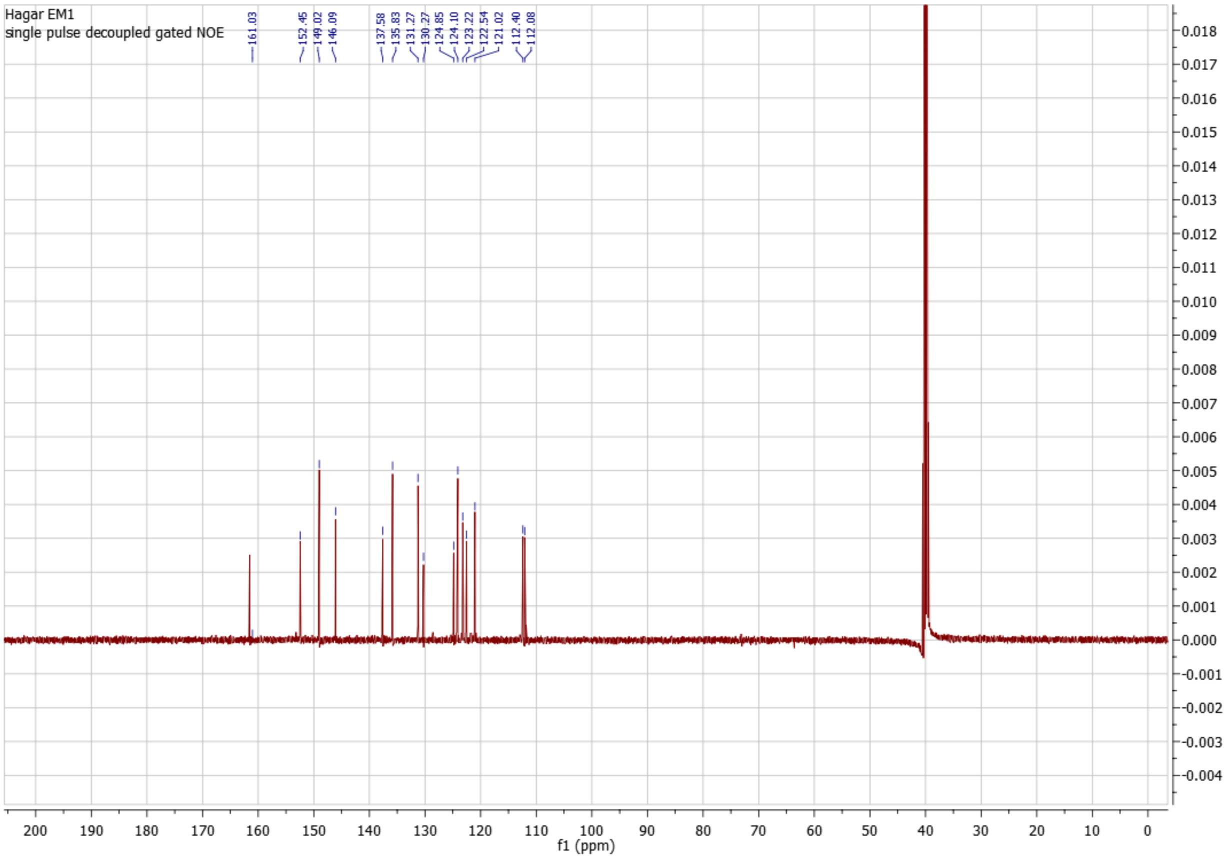

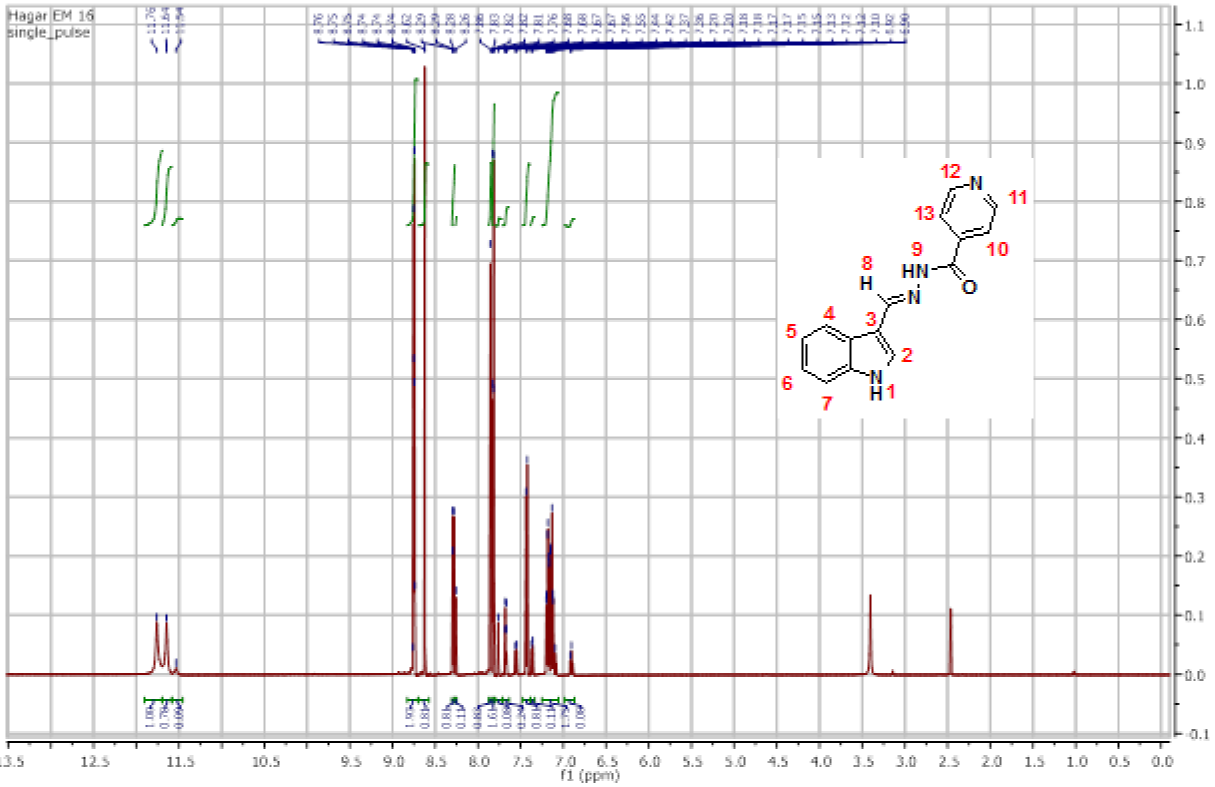

Figure S4 ^{1}H NMR spectrum of **3b**.

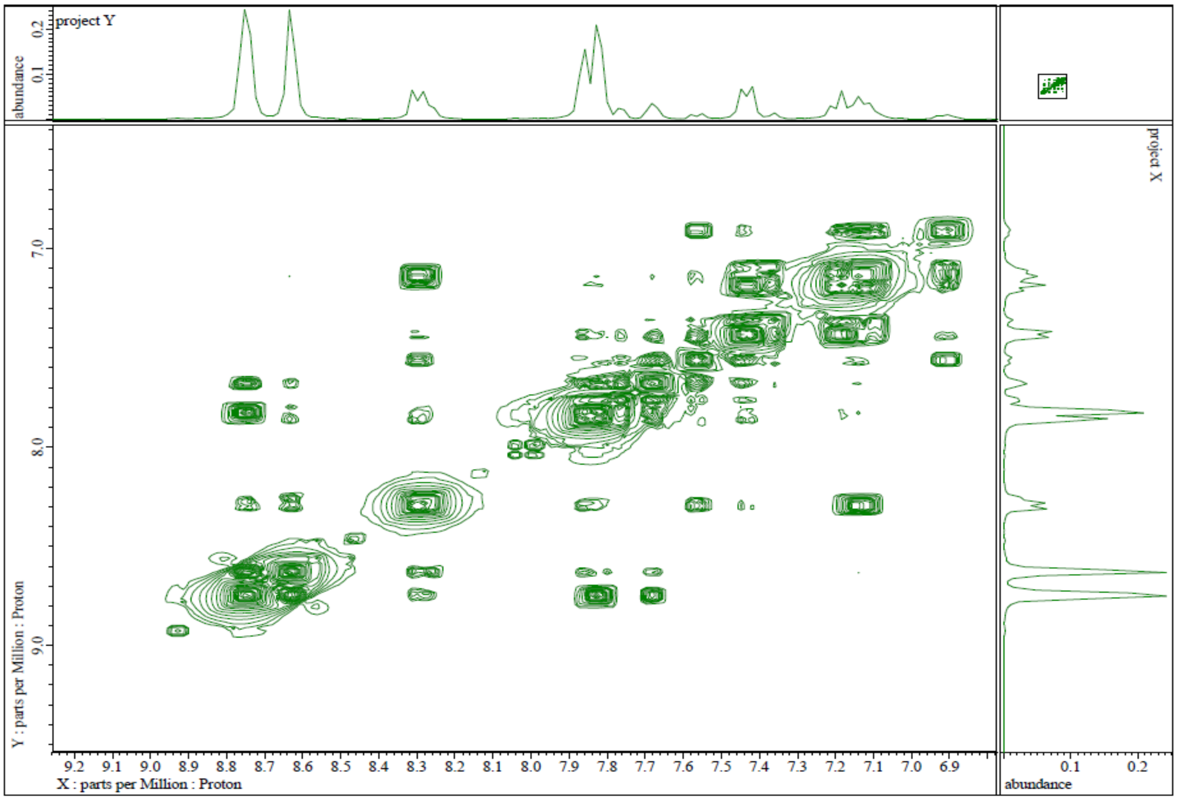

Figure S5 COSY spectrum of **3b**.

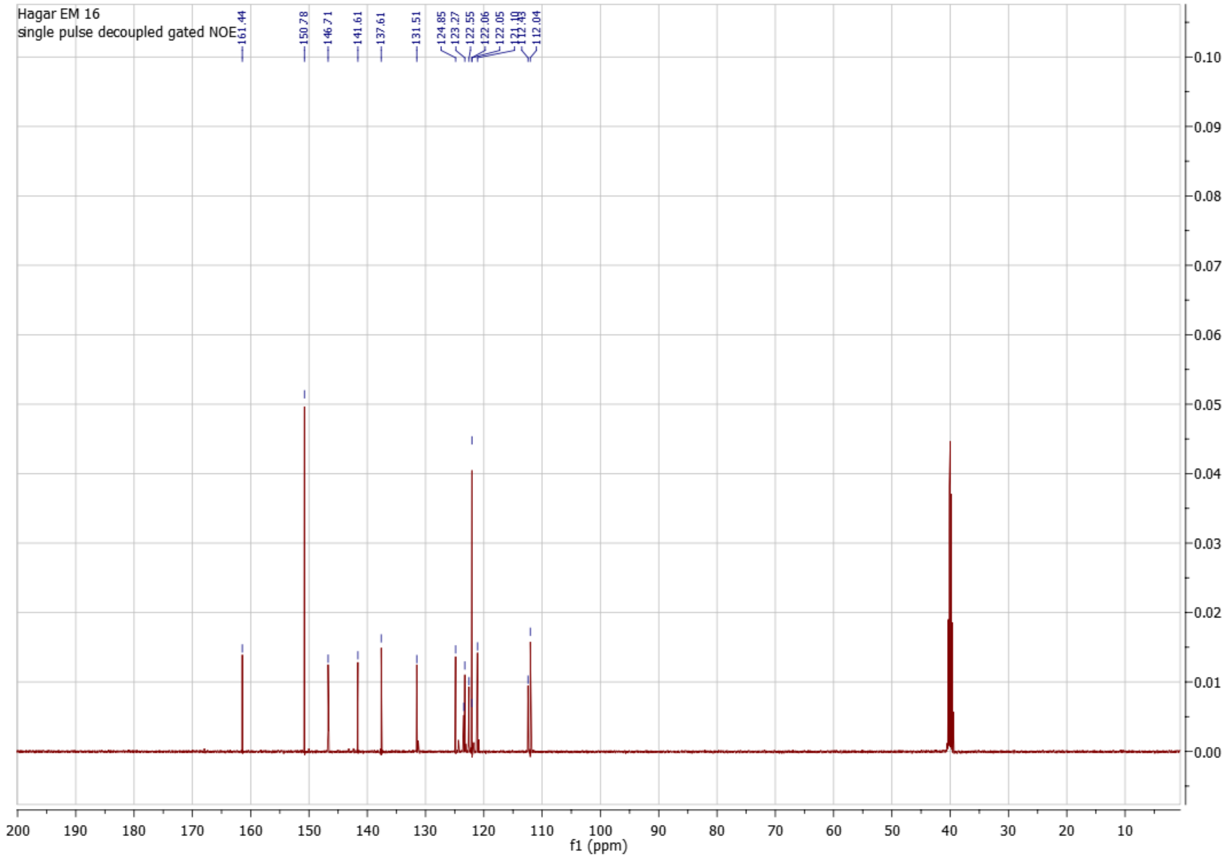

Figure S6 ^{13}C NMR spectrum of **3b**.

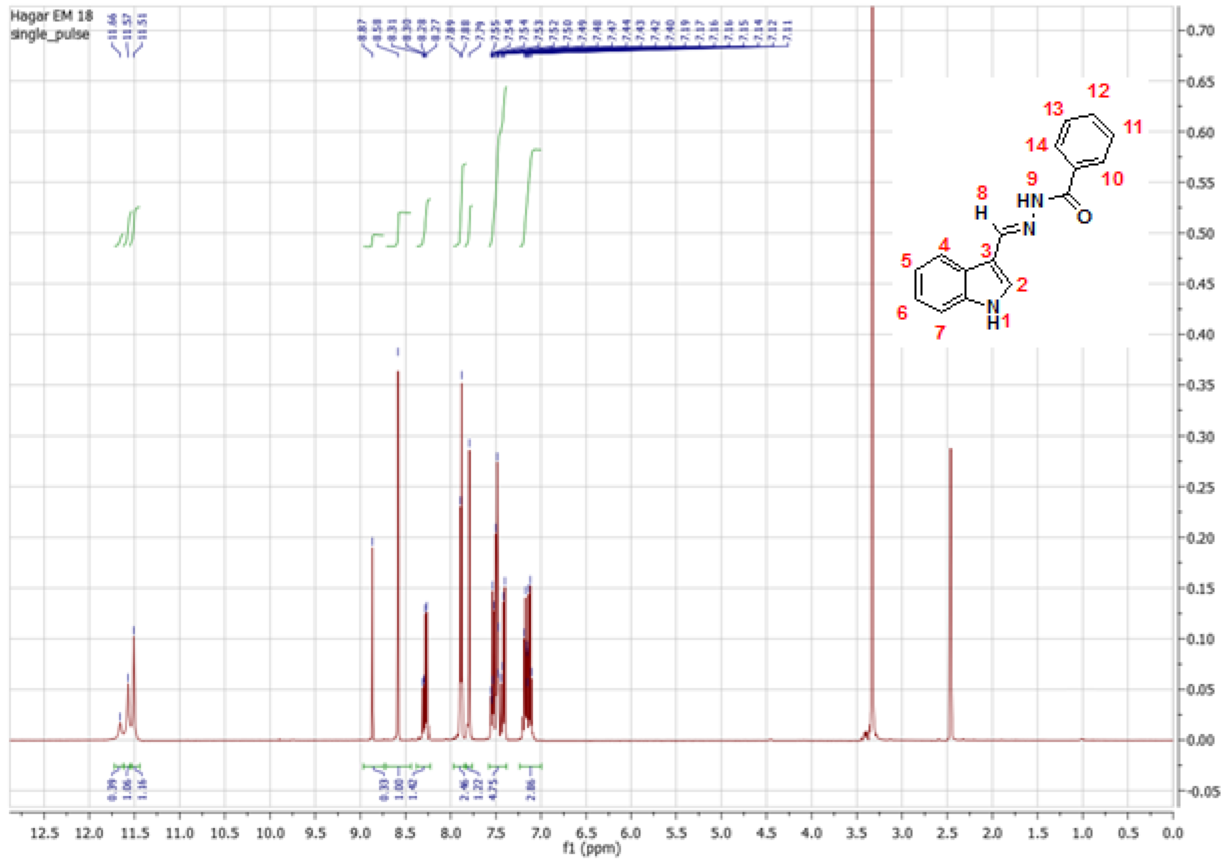

Figure S7 ^{1}H NMR spectrum of **3c**.

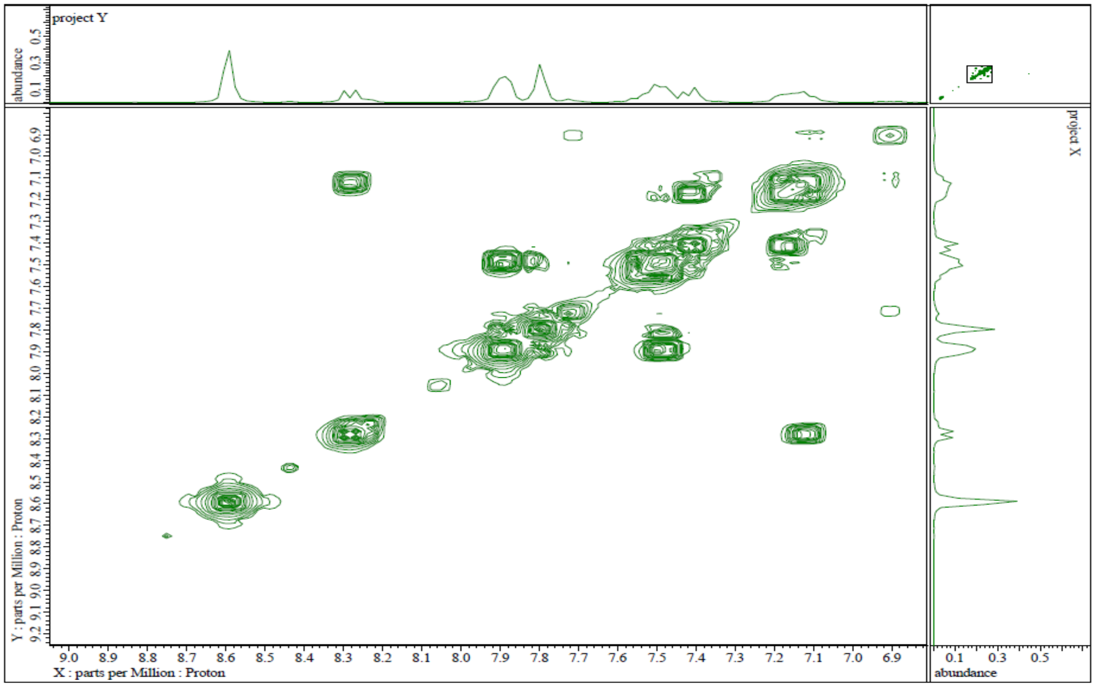

Figure S8 COSY spectrum of **3c**.

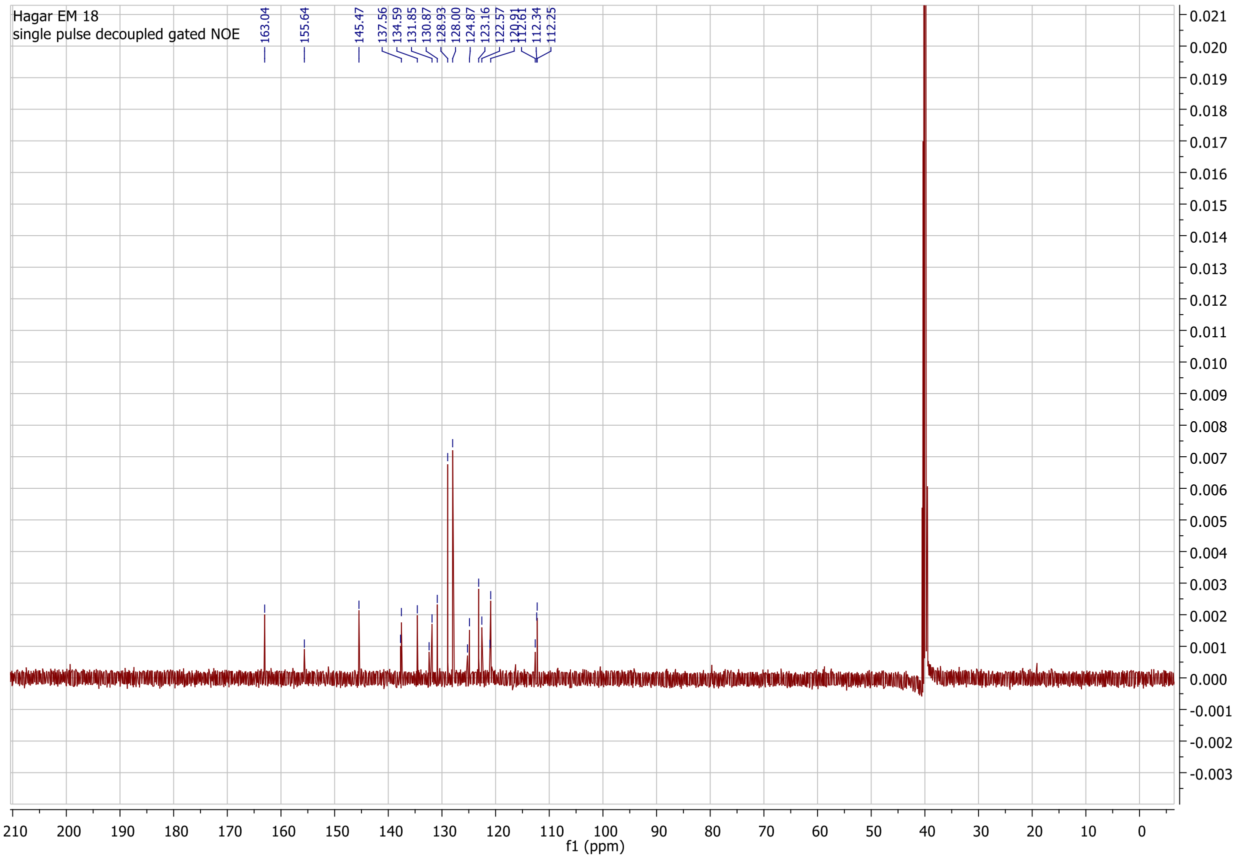

Figure S9 ^{13}C NMR spectruma of **3c**.

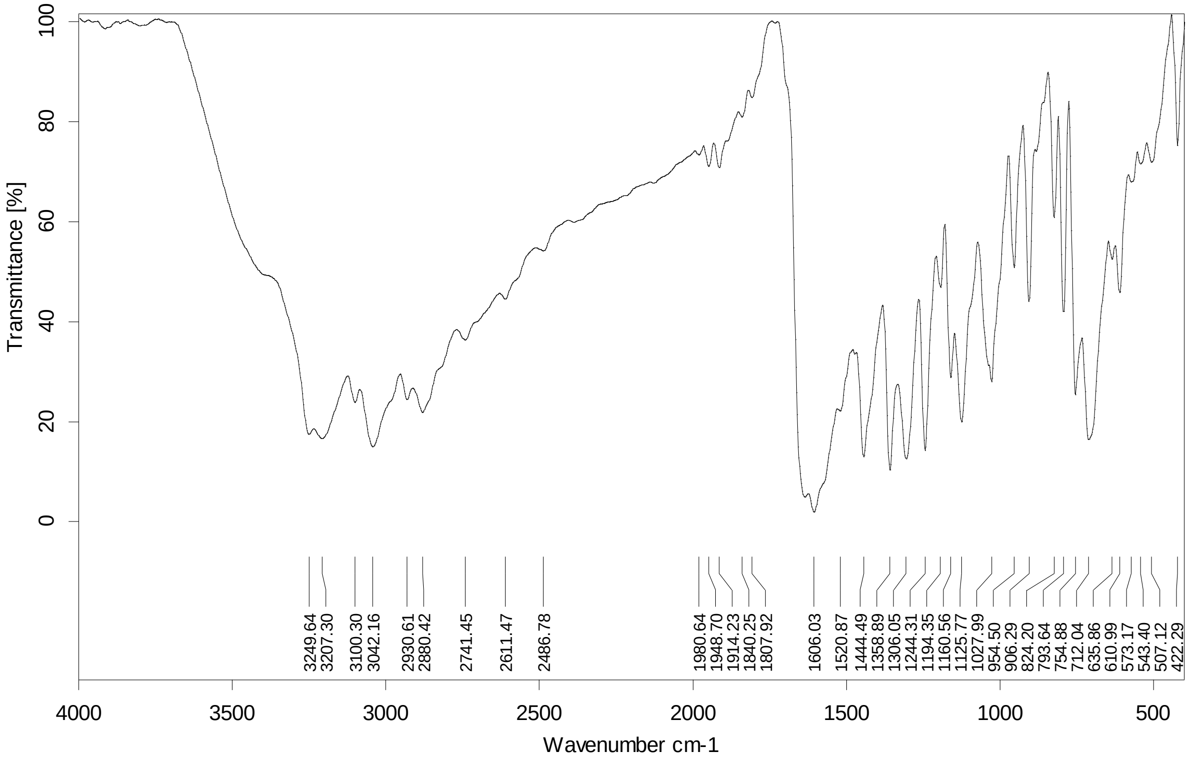

Figure S10 FTIR spectra of **3a**.

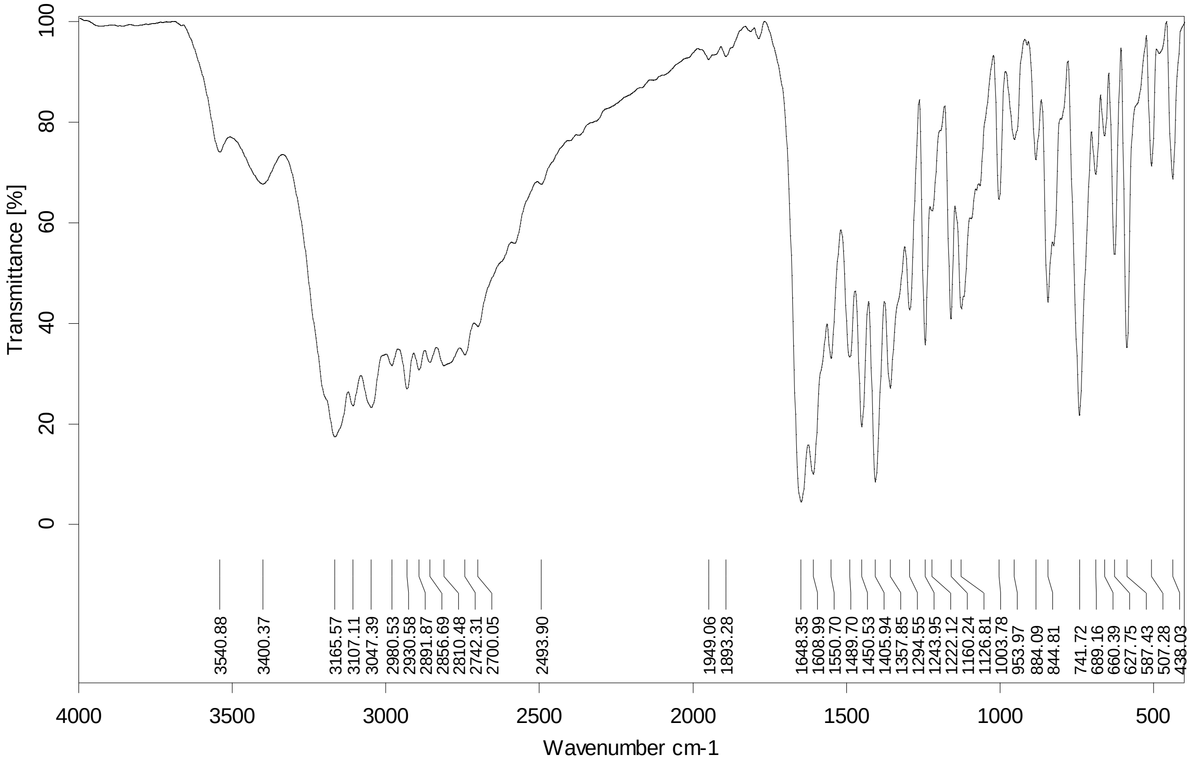

Figure S11 FTIR Spectra of **3b**.

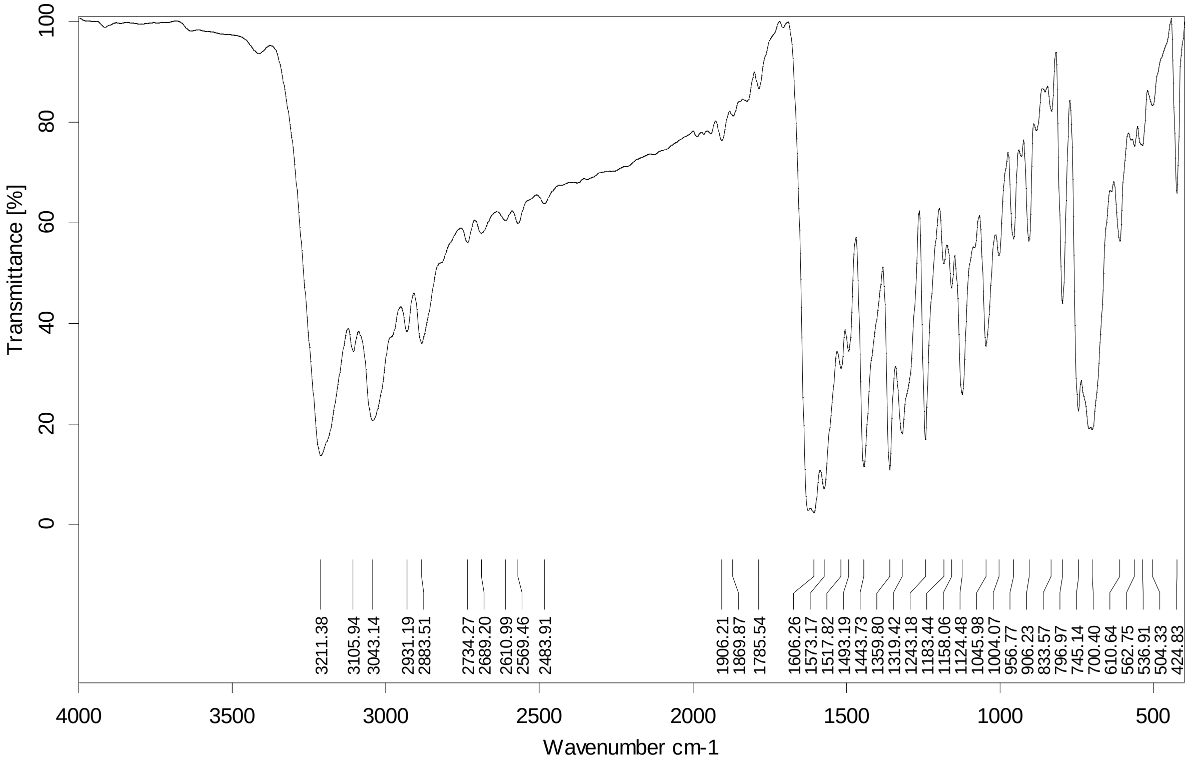

Figure S12 FTIR spectra of **3c**.
